# Characterization of a novel aromatic substrate-processing microcompartment in Actinobacteria

**DOI:** 10.1128/mbio.01216-23

**Published:** 2023-07-18

**Authors:** Lior Doron, Markus Sutter, Cheryl A. Kerfeld

**Affiliations:** 1 MSU-DOE Plant Research Laboratory, Michigan State University, East Lansing, Michigan, USA; 2 Environmental Genomics and Systems Biology Division, Lawrence Berkeley National Laboratory, Berkeley, California, USA; 3 Molecular Biophysics and Integrative Bioimaging Division, Lawrence Berkeley National Laboratory, Berkeley, California, USA; 4 Department of Biochemistry and Molecular Biology, Michigan State University, East Lansing, Michigan, USA; University of California, Berkeley, Berkeley, California, USA

**Keywords:** metabolosomes, 2-aminophenol meta cleavage pathway, 2-AP 1,6-dioxygenase, *Micromonospora rosaria*

## Abstract

We have discovered a new cluster of genes that is found exclusively in the Actinobacteria phylum. This locus includes genes for the 2-aminophenol *meta*-cleavage pathway and the shell proteins of a bacterial microcompartment (BMC) and has been named aromatics (ARO) for its putative role in the breakdown of aromatic compounds. In this study, we provide details about the distribution and composition of the ARO BMC locus and conduct phylogenetic, structural, and functional analyses of the first two enzymes in the catabolic pathway: a unique 2-aminophenol dioxygenase, which is exclusively found alongside BMC shell genes in Actinobacteria, and a semialdehyde dehydrogenase, which works downstream of the dioxygenase. Genomic analysis reveals variations in the complexity of the ARO loci across different orders. Some loci are simple, containing shell proteins and enzymes for the initial steps of the catabolic pathway, while others are extensive, encompassing all the necessary genes for the complete breakdown of 2-aminophenol into pyruvate and acetyl-CoA. Furthermore, our analysis uncovers two subtypes of ARO BMC that likely degrade either 2-aminophenol or catechol, depending on the presence of a pathway-specific gene within the ARO locus. The precise precursor of 2-aminophenol, which serves as the initial substrate and/or inducer for the ARO pathway, remains unknown, as our model organism *Micromonospora rosaria* cannot utilize 2-aminophenol as its sole energy source. However, using enzymatic assays, we demonstrate the dioxygenase’s ability to cleave both 2-aminophenol and catechol *in vitro*, in collaboration with the aldehyde dehydrogenase, to facilitate the rapid conversion of these unstable and toxic intermediates.

**IMPORTANCE:**

Bacterial microcompartments (BMCs) are proteinaceous organelles that are widespread among bacteria and provide a competitive advantage in specific environmental niches. Studies have shown that the genetic information necessary to form functional BMCs is encoded in loci that contain genes encoding shell proteins and the enzymatic core. This allows the bioinformatic discovery of BMCs with novel functions and expands our understanding of the metabolic diversity of BMCs. ARO loci, found only in Actinobacteria, contain genes encoding for phylogenetically remote shell proteins and homologs of the *meta*-cleavage degradation pathway enzymes that were shown to convert central aromatic intermediates into pyruvate and acetyl-CoA in gamma Proteobacteria. By analyzing the gene composition of ARO BMC loci and characterizing two core enzymes phylogenetically, structurally, and functionally, we provide an initial functional characterization of the ARO BMC, the most unusual BMC identified to date, distinctive among the repertoire of studied BMCs.

## INTRODUCTION

**Fig 1:**
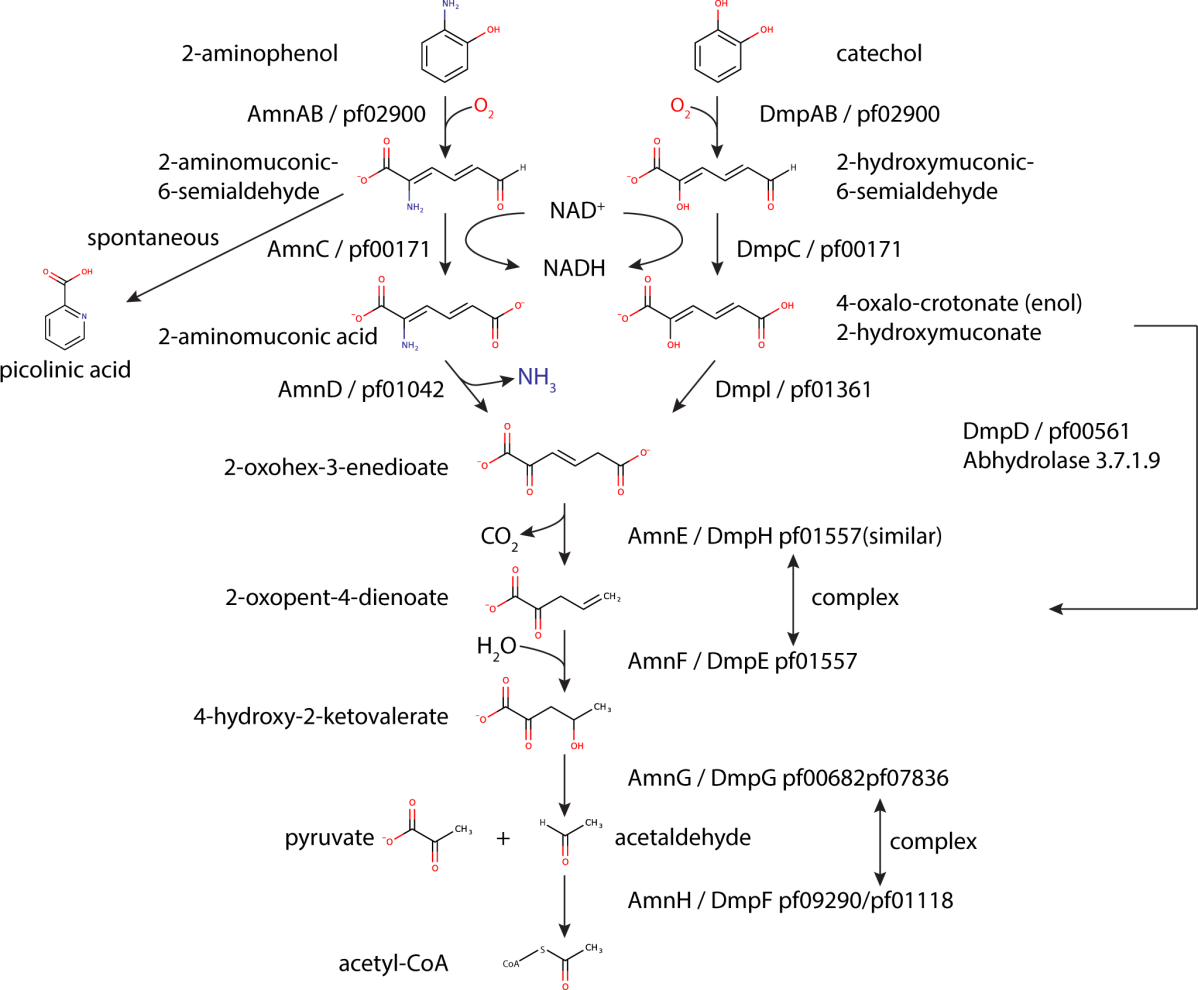
2-Aminophenol and catechol degradation pathway leading to the formation of pyruvate and acetyl-CoA. Enzyme names from known pathways such as those found in *Pseudomonas putida* (Dmp) and *Comamonas testosteroni* (Amn) are indicated. Black arrows mark the catabolic enzymes (left scheme, 2-aminophenol pathway [AmnAB, 2-aminophenol-1,6-dioxygenase; AmnC, 2-aminomuconic-6-semialdehyde dehydrogenase; AmnD, 2-aminomuconate deaminase]; right scheme, catechol pathway [DmpAB, catechol 2,3-dioxygenase; DmpC, 2-hydroxymuconic semialdehyde dehydrogenase; DmpD, 2-hydroxymuconate-semialdehyde hydrolase; DmpI, 2-hydroxymuconate tautomerase]; center scheme [AmnE/DmpH/XylI, 4-oxalocrotonic acid decarboxylase; AmnF/DmpE/XylJ, 2-keto-4-pentenoate hydratase; AmnG/DmpG/XylK, 4-hydroxy-2-oxovalerate aldolase; AmnH/DmpF, acetaldehyde dehydrogenase]).

Many microorganisms are enriched in pathways for the catabolism of different aromatic substrates, which can then be used as a carbon and/or nitrogen source (1). In bacteria, the biodegradation of aromatic substrates has been extensively studied and is divided into two main pathways. The peripheral or upper pathways transform many structurally diverse aromatic substrates through a wide variety of catabolic pathways into a few central intermediates. These, in turn, are broken down into metabolites such as acetyl-CoA, succinyl-CoA, and pyruvate by pathways that are functionally and evolutionarily conserved (2). The catechol and 2-aminophenol (2-AP) *meta*-cleavage pathways are analogous and are encoded by very similar sets of genes (Fig. 1). Both pathways start with de-aromatization and cleavage of the benzene ring by a reaction that is catalyzed by an extradiol-type dioxygenase that uses Fe(III) as a cofactor (3). The ring cleavage of catechol is catalyzed by catechol 2,3-dioxygenase (DmpAB) (4), which cleaves the carbon–carbon bond adjacent to the phenolic hydroxyl groups. In contrast, the 2-AP ring cleavage is catalyzed by 2-AP 1,6-dioxygenase (AmnAB) (5), which oxidizes substrates with an amino group adjacent to a hydroxyl group. Notably, both the dioxygenase and the downstream enzyme in each pathway, 2-amino/2-hydroxymuconinc semialdehyde dehydrogenase, demonstrate promiscuity toward each other’s substrates, with preference toward the physiological substrate. However, in the case of 2-AP 1,6-dioxygenase, it was demonstrated that the catechol oxidation product, 2-hydroxymuconic semialdehyde, inhibited enzymatic activity (6 - 9). Although the 2-AP and catechol *meta*-cleavage pathways start differently, they converge at the 2-oxohex-3-enedioate intermediate (10). This is formed by a deamination reaction by 2-aminomuconate deaminase (AmnD) (11), or a tautomerization by 2-hydroxymuconate tautomerase (DmpI; also referred to as 4-oxalocrotonate tautomerase) (12 - 14) for the 2-AP or catechol pathways, respectively. Both the deaminase and the tautomerase can only catalyze the reaction of their physiological substrates; thus, their genes are defined as pathway specific.

The enzymes involved in catabolism of aromatic compounds have, to date, all been assumed to be cytosolically localized. However, a bioinformatic catalog of bacterial microcompartments (BMCs) recently identified a new type of bacterial metabolic organelle confined to the phylum Actinobacteria that was proposed to be involved in the degradation of aromatic aldehyde compounds and so named the ARO BMC (15). The locus contains genes for shell proteins which form the delimiting “membranes” of BMCs and for homologs of the 2-AP catabolic pathway, which are found in close proximity to an IclR-type transcriptional regulator, suggestive of an operon-like structure (Fig. S1A).

**Fig 2:**
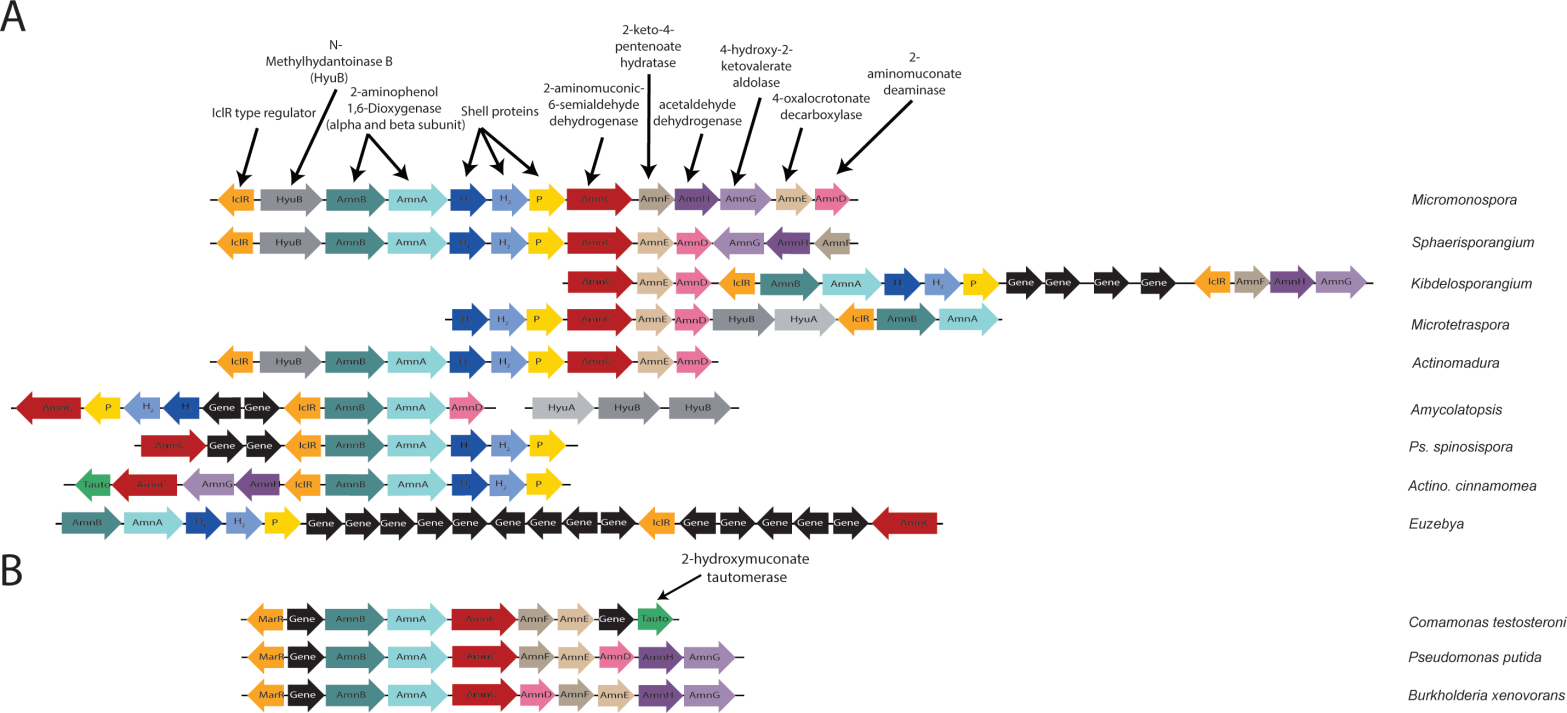
ARO loci in representatives of Actinobacteria. 2-AP/catechol locus diagrams of ARO BMC-encoding species (**A**) or non-ARO nitrobenzene-degrading species (**B**). Non-ARO pathway genes are colored black.

In general, despite different substrates and diverse functions, catabolic BMCs, also known as metabolosomes, share a common enzymatic core composed of a substrate-defining signature enzyme that generates an aldehyde, and an aldehyde dehydrogenase (AldDh; pfam00171) that uses both CoA and NAD^+^ as cofactors and disarms the toxic aldehyde intermediate (16). However, several observations set ARO BMCs apart from all other metabolosomes. First, based on the co-occurence of a putative 2-AP 1,6-dioxygenase, which is suggested to catalyze the benzene ring cleavage of 2-AP (herein referred to as ARO dioxygenase), with shell proteins, the ARO BMC is the first metabolosome predicted to be involved in the degradation of aromatic substrates. Second, unlike all other BMC-associated AldDhs, which are targeted to the interior of shells via encapsulation peptide (EP) extensions (17, 18), the ARO 2-aminomuconic-6-semialdehyde dehydrogenase homolog (herein referred as ARO AldDh) does not contain a detectable EP nor does it belong to the family of CoA-dependent AldDh. Therefore, the ARO AldDh is an outlier on the phylogenetic tree of all the known BMC-associated aldehyde dehydrogenases, isolated on a lone branch (15). In addition to the unique enzymes, the shell, the delimiting “membrane” of the ARO BMC, is likewise unusual. It is very simple, made up of three shell proteins: one BMC-P gene product and two distinct types of BMC-H proteins; in contrast, typical BMC shell facets contain three to five copies of a combination of BMC-H and BMC-T gene products (19). While one of the ARO BMC-H shell proteins (H_cerulean) is predicted to form a canonical hexamer with the typical “P(R/K)PH” motif, which mediates one of the main inter-hexamer lateral interactions (20), the other hexamer (H_lipstickred) is on an isolated branch relative to all other known shell proteins (see Fig. 2B in reference 21)). Phylogenetic analysis of all BMC shell proteins showed that the ARO shell proteins are all found in late-branching subclades, suggesting that compared to other shell proteins, the ARO shell proteins have evolved to a distinct function, probably related to the aromatic substrates (15). The novel substrate for a BMC, the simple shell made of remotely branching proteins, as well as the restricted phylogenetic occurrence, indicates that the ARO is possibly a highly specialized metabolic organelle. Relative to all other functionally characterized BMCs, the composition of the shell proteins and the function of enzymatic core of the ARO BMC are unusual, perhaps explaining its confinement to the Actinobacteria. The fact that the enzymatic pathway for the degradation of 2-AP is otherwise not associated with BMCs, makes the ARO BMC a unique system to explore.

Here, we present a bioinformatic characterization of ARO loci found across the Actinobacteria and provide a phylogenetic, functional, and structural characterization of the ring-opening ARO dioxygenase and its downstream partner in the pathway, ARO AldDh. By comparing ARO BMC loci from representatives across the phylum Actinobacteria and by performing phylogenetic analyses of ARO dioxygenase and ARO AldDh, we substantiate the proposed encapsulation of ARO dioxygenase as the signature enzyme in ARO BMC. By solving the crystal structure of the ARO dioxygenase from *Micromonospora rosaria,* we demonstrated the similarity of the enzyme to a known 2-AP 1,6-dioxygenase. Furthermore, we identify two ARO BMC loci subtypes that differ in only one gene that determines whether the initial substrate is 2-AP or catechol, suggesting that depending on the species, ARO BMCs are most probably involved in the degradation of either 2-AP or catechol. We further show that in contrast to the characterized 2-AP-degrading Proteobacteria, *M. rosaria*, an ARO BMC locus-containing species, cannot grow on 2-AP as a sole energy source, thus providing evidence that 2-AP does not likely induce the formation of the ARO BMC; therefore, the identity of the substrate that is converted into 2-AP is yet to be discovered. Finally, to demonstrate the involvement of ARO enzymes in the initial attack on 2-AP *in vitro*, we expressed and purified the recombinant ARO dioxygenase and ARO AldDh from *M. rosaria* and subjected them to single and coupled enzyme activity assays. Our results show the ability of recombinant ARO dioxygenase to interact with either catechol or 2-AP substrates and convert them into aldehyde intermediates, which are then detoxified by the ARO AldDh. Collectively, this study provides an initial functional characterization of the ARO BMC and sheds light on the requirement for BMC encapsulation of 2-AP pathway enzymes in Actinobacteria.

## RESULTS

### Bioinformatic analysis of the ARO BMC locus

A BLAST search with the sequence of ARO BMC-H_lipstickred against 64,495 isolate genomes available from the Integrated Microbial Genomes database (IMG; img.jgi.doe.gov) identified homologs with high identity (>55%) in 33 species from the phylum Actinobacteria. All of the homologs were found proximal to other ARO shell proteins and an IclR-type regulator, verified by comparing them to established ARO BMC protein sequences (15). A phylogenetic tree analysis based on complete 16S rRNA gene sequences from ARO BMC locus-containing species revealed that the ARO BMC-encoding species cluster into three subclades, with one subclade belonging to the *Micromonosporales* order, the second to the *Pseudonocardiales* order, and the third to the *Streptosporangiales* order of the Actinomycetia class. Two species, *Euzebya tangerina* F10 and *Streptomyces* sp. AmelKG-F2B of the Nitriliruptoria and Actinomycetia classes, respectively, were outliers in the phylogenetic tree, found on a separate branch (Fig. S1B). We identified all the homologs of 2-AP pathway genes and neighboring genes that were conserved across bacterial orders and built a data set of all the ARO BMC loci (a table with all IMG gene IDs is available at https://www.kerfeldlab.org/aro_supplementary.html).

We focused on the *Micromonospora* genus which comprises the majority of the ARO BMC-encoding species. A 16S rRNA phylogenetic tree analysis of representative of the *Micromonospora* genus shows that the ARO BMC-associated organisms are not confined to one specific branch but instead were found in different branches of the tree and cluster with other species that were shown to occupy different niches, such as soil and aquatic sediments (Fig. S1C). A comparison of the ARO loci in the *Micromonospora* genus and in representatives of different Actinobacterial orders revealed variations in its complexity, ranging from a very simple main locus with shell proteins and enzymes of only the initial reactions steps up to an extensive locus that contains all the genes that are required for the breakdown of 2-AP into the central metabolites pyruvate and acetyl-CoA in a single operon (as defined by those found in Micromonosporaceae ARO BMC-encoding species) (Fig. 2A; Fig. S2). This lack of conservation of genetic organization of the pathway genes contrasts with non-BMC-associated pathways of the Proteobacteria, which is often found as a complete operon (22) (Fig. 2B).

Our analysis revealed that a compact gene arrangement of all the structural and enzymatic core genes in a single operon is found in species of the *Micromonospora* and *Verrucosispora* genera of the *Micromonosporales* order and in *Sphaerisporangium rubeum* DSM 44936 of the *Streptosporangiales* order (Fig. 2; Fig. S2). Although these species are not located on the same branch in the 16S rRNA phylogenetic tree, they all share the genomic region that is found upstream of the IclR-type regulator. Those sets of genes were also found in *Actinomadura viridis* DSM 43175 of the *Streptosporangiales* order but not in ARO BMC-encoding species of the *Pseudonocardiales* order, suggestive of a horizontal gene transfer of the ARO BMC gene cluster from the *Streptosporangiales* order to the *Micromonosporales* order. Among the genes that were found directly upstream to the ARO BMC locus, we identified the gene HyuA that encodes subunit A of a protein annotated as a methylhydantoinase with unknown function. Its partner, HyuB, was usually found within the ARO operon (Fig. S2). Their role is not clear nor can it be established if the enzyme is encapsulated in the ARO BMC because it is not part of the canonical 2-AP degradation pathway in non-BMC-containing species. The comparison also revealed that the gene for 2-aminomuconate deaminase, which removes the amine group of 2-aminomuconic acid is missing from the ARO locus in some species. Instead, in these organisms, we identified a homolog for 2-hydroxymuconate tautomerase, which is required for the catechol *meta-*degradation pathway. The identification of two ARO BMC subtypes indicates that depending on the species, the ARO BMCs have specialized to process either 2-AP or catechol from a yet-to-be-identified upper pathway.

**Fig 3:**
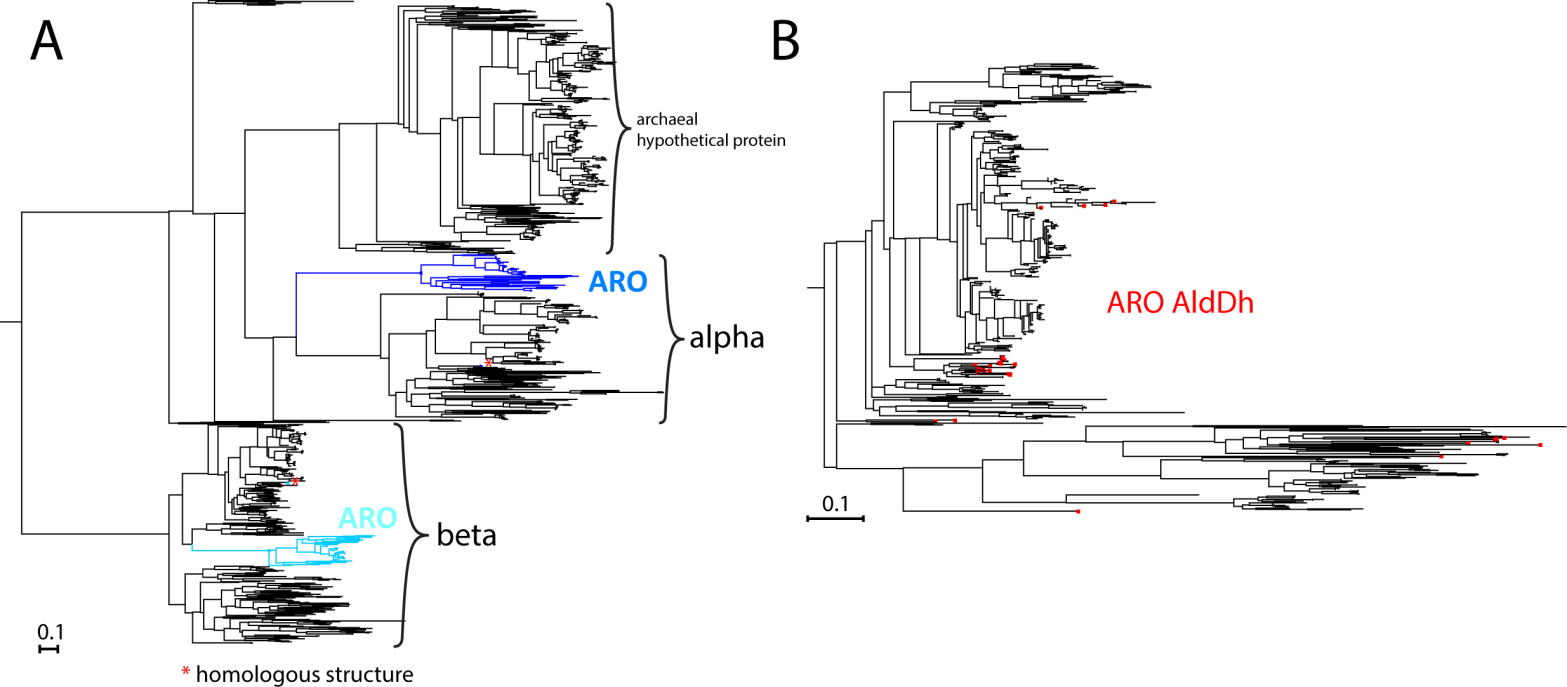
Phylogenetic trees of ARO dioxygenase subunits and aldehyde dehydrogenase. (**A**) Phylogenetic tree of ARO dioxygenase and its homologs from a BLAST search against all genomes. Positions of dioxygenases from ARO loci from Actinobacteria are colored (alpha subunit in blue, beta subunit in cyan), and the homologs from *Comamonas testosteroni* for which there is a crystal structure are marked with an asterisk. (**B**) Phylogenetic tree of homologs of the ARO aldehyde dehydrogenase from a BLAST search against all Actinomycete genomes. Positions of the ARO AldDh are colored red.

Despite some variations in gene order, we find several patterns. First, in most of the ARO loci-containing species, the two ARO dioxygenase subunit genes were located immediately upstream of the shell proteins, which typically correlates with encapsulation. Indeed, a sequence alignment of homologs of the ARO dioxygenase beta subunit across bacteria of different phyla (see Supplementary Data Set 1) identified an N-terminal region, of 30–35 residues, that is found only in ARO BMC-encoding strains and may serve as a determinant for encapsulation (Fig. S3A). However, unlike canonical EPs (17, 23), the extension is not predicted to be alpha helical. Furthermore, when searching for the closest homologs to the ARO dioxygenase we were unable to find any actinobacterial hits that were not part of an ARO locus, suggesting that this pathway is always encapsulated in Actinobacteria. A phylogenetic tree of the alpha and beta subunits of the ARO dioxygenase reveals that the BMC-associated ARO dioxygenase subunits are unique and form separate branches from homologs found in other bacteria (Fig. 3A). This type of occurrence and clustering was not observed for ARO AldDh (Fig. 3B), suggesting that the AldDh may also be used outside of the BMC in Actinobacteria, whereas the ARO dioxygenase needs to be encapsulated in the ARO BMC. We also noticed that the genes for the enzymes that are involved in the early stages of the *meta-*cleavage pathway (e.g., ARO dioxygenase, ARO AldDh, 2-aminomuconate deaminase, or 2-hydroxymuconate tautomerase) often co-occurred with the genes for the shell proteins in the ARO locus. In contrast, the genes for the enzymes of the later stages (e.g., 2-keto-4-pentenoate hydratase, 4-oxalocrotonate decarboxylase, acetaldehyde dehydrogenase, and 4-hydroxy-2-ketovalerate aldolase) were absent from the ARO locus in many ARO BMC-encoding species (Fig. S2). These genes were usually found adjacent to each other in other regions of the genome, in accordance with the ability of 2-keto-4-pentenoate hydratase and 4-oxalocrotonate decarboxylase on the one hand, or acetaldehyde dehydrogenase and 4-hydroxy-2-ketovalerate aldolase on the other, to form bifunctional complexes (24 - 28) (Fig. S2). Whether these enzymes interact with the enzymes expressed from the ARO locus, however, is yet not known.

### Characterizing the physiological role of the ARO BMC

To determine if 2-AP is the physiological substrate of ARO BMC from the *Micromonospora* and *Verrucosispora* genera of the *Micromonosporales* order, we acquired the *M. rosaria* strain DSM 803 from the German Collection of Microorganisms and Cell Cultures GmbH and monitored its ability to utilize 2-AP as a carbon or nitrogen source. No growth was observed when 2-AP was added to a minimal media that lacks either glucose or NH_4_ (Fig. S4A), demonstrating the inability of *M. rosaria* to exploit 2-AP in the same manner that was previously shown for various nitrobenzene degrading bacteria (5, 22). We note that growth was observed on rich media supplemented with 2-AP at a concentration of 100 µM and lower; however, higher concentrations of 2-AP were toxic to the cells (Fig. S4B). The toxicity of 2-AP despite the BMC-associated pathway to degrade it to downstream products suggests that the BMC locus is not induced by the presence of 2-AP in the medium. The initial substrate of the pathway and the inducer for transcription of the BMC locus, therefore, is not 2-AP but likely an upstream precursor of it.

### Structure of the ARO dioxygenase

To structurally and functionally characterize the ARO dioxygenase and to demonstrate its ability to degrade 2-AP, we recombinantly co-expressed and purified the alpha and a His-tagged beta subunit (ARO AmnA and AmnB) from *Micromonospora rosaria* (29). We detected two bands corresponding to the alpha and beta subunits of ARO dioxygenase on SDS-PAGE of the HisTrap eluate (Fig. S5A). The co-elution of the subunits and the observation that neither of the subunits was soluble when expressed on their own (Fig. S5B), suggest a requirement for an interaction between the two subunits during folding. This interaction was verified by size exclusion chromatography analysis, which yielded a major peak at 140 kDa, corresponding to a tetramer composed of two heterodimers, as observed for other dioxygenases (7, 30) (Fig. S5C).

**Fig 4:**
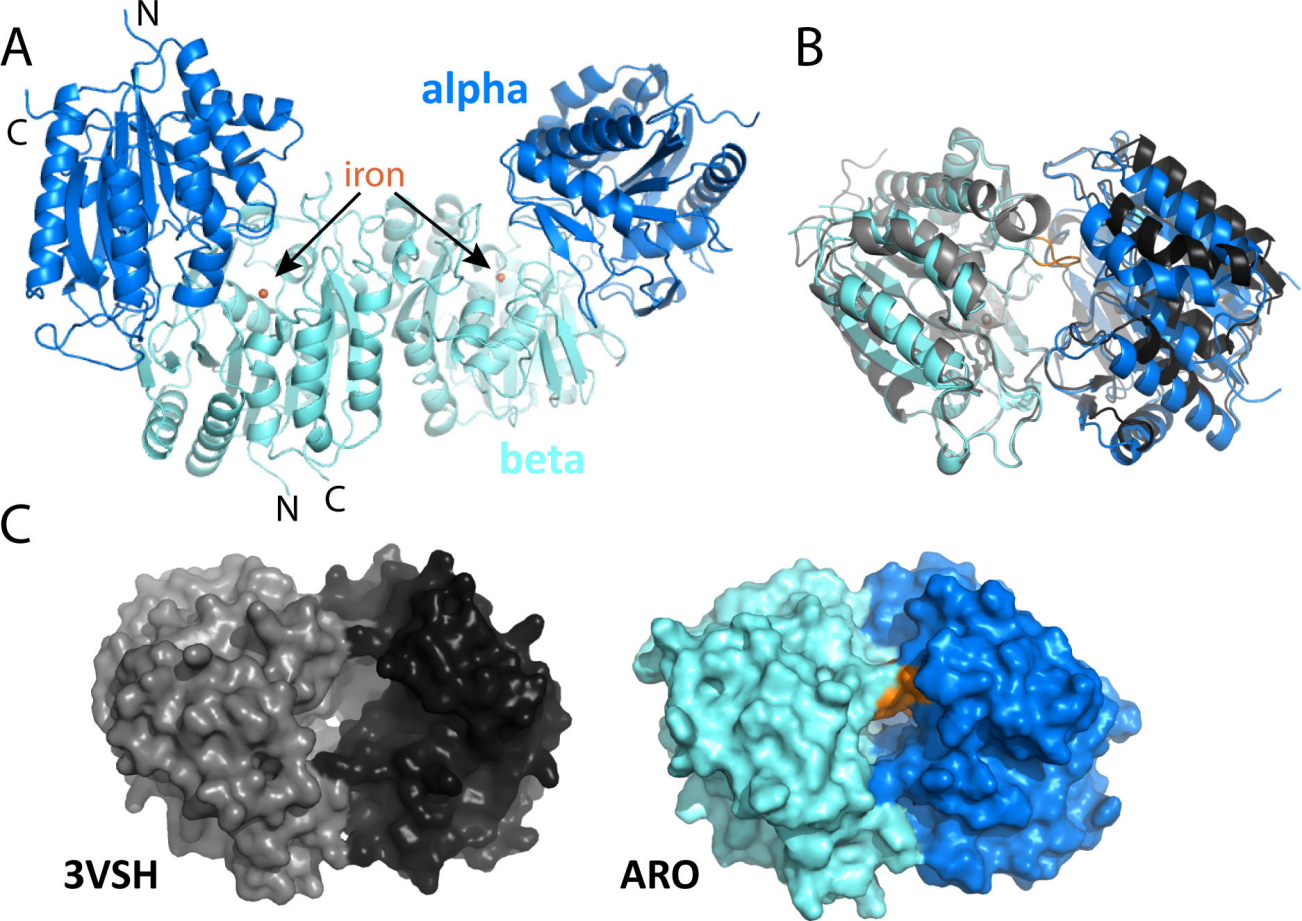
Structure overview of *M. rosaria* ARO dioxygenase and comparison with the non-BMC-associated homolog from *Comamonas testosteroni*. (**A**) Structure of the biological dimer with iron active site in the beta subunit marked. N-terminal and C-terminal residues (D3 and H278, respectively, for alpha; T30 and G322 for beta subunits) are marked. (**B**) Alignment of ARO dioxygenase with the homologous structure (with aligned beta subunits); a large difference is observed in a loop at residues N184-Y189. (**C**) Comparison of surface view of the two structures reveals differences in active site accessibility.

We determined the crystal structure of the ARO dioxygenase from *Micromonospora rosaria* at 1.75 Å resolution. Data collection and refinement statistics are found in Table S1. Two alpha/beta heterodimers form a biological dimer (Fig. 4A) via their beta subunits, consistent with our size exclusion analysis, and we find two of those in the asymmetric unit of the P1 unit cell. We can model residues D3 to H278 of the alpha subunit and T30 to G322 of the beta subunit, as well as the iron atom in the active site into the electron density. The lack of density for the first 29 amino acids for the beta subunit is consistent with the possibility that this extension is involved in encapsulation; in structures of BMC-associated enzymes with EPs, the extensions are typically not visible in the electron density, as exemplified in the crystal structures of both PDU1C-associated flavodoxin and the GRM3-associated AldDh (31 - 33). The structure superimposes on a non-BMC-associated homolog (PDB ID: 3VSH) with an rmsd of 1.46 Å over 485 aligned Cα atoms for the whole enzyme, 0.99 Å over 232 aligned Cα atoms for the alpha subunit, and 0.56 Å over 251 Cα atoms for the beta subunit. However, one large difference between the ARO BMC-associated dioxygenase and its non-BMC-associated counterpart is in a loop close to the active site for the beta subunit involving residues N184-Y188 (Fig. 4B, orange). A consequence of the difference is a much more shielded active site as seen in the surface representation (Fig. 4C).

### Functional characterization of ARO dioxygenase

**Fig 5:**
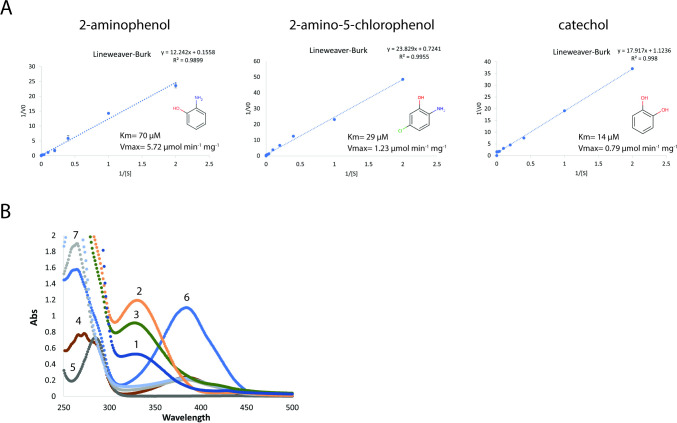
*In vitro* functional characterization of the ARO dioxygenase and the ARO AldDh. (**A**) Lineweaver–Burke plots of ARO dioxygenase with 2-AP (left panel), 2-amino-5-chlorophenol (middle panel), and catechol (right panel). The reaction mixture (1 mL) contained 10 mM sodium phosphate buffer (pH 8.0) and 27 µg enzyme. Each reaction was started by adding the specific substrate. (**B**) Conversion of 2-AP (λ_max_ at 282 nm) to 2-aminomuconate (λ_max_ at 326 nm) in a coupled enzyme assay using ARO dioxygenase and ARO AldDh. Curves represent ARO AldDh activity from total soluble proteins (curve 1) after ammonium sulfate precipitation (40%, curve 2) and after HIC-phenyl chromatography (fraction 32, curve 3). Reactions lacking NAD^+^ (curve 4), or ARO dioxygenase (curve 5), were used as control experiments. The spontaneous conversion of 2-AMS (λ_max_ at 380 nm) to picolinic acid (λ_max_ at 263 nm) in the absence of ARO AldDh is depicted in curves 6 (time 0) and 7 (10 minutes). The reaction mixture (1 mL) contained 100 mM potassium phosphate (pH 7.5), 0.5 µM 2-AP, 27 µg of purified dioxygenase, 20 µg of purified ARO AldDh, and 20 µM NAD^+^. The reaction was started by adding 2-AP. Results were obtained from three independent replicate experiments.

We determined the ability of ARO dioxygenase to catalyze the ring cleavage of 2-AP, 2-amino-5-chlorophenol (2A5CP), and catechol *in vitro*. The *V*max and *K*m were calculated from Lineweaver–Burke plots and are shown in Fig. 5A and correspond to a specific maximal turnover rate (*Kcat*) of 277 s^−1^, 59 s^−1^, and 38 s^−1^, for 2-AP, 2A5CP, and catechol, respectively. These results indicate that in addition to utilizing 2-AP (100% relative activity), ARO dioxygenase also utilized 2A5CP (21% activity) and catechol (13% activity) as substrates, although its catalytic efficiency was one order of magnitude lower than those reported previously for 2-AP 1,6-dioxygenase from non-ARO BMC-encoding species (5, 6, 30). The ring cleavage of 2-AP and 2A5CP results in unstable products, as observed by the decrease of A_380_, similar to those observed for 2-aminomuconic acid semialdehyde, which was shown to spontaneously convert to picolinic acid (10, 34) (Fig. S6; Fig. 1). We note that catechol exhibited a fivefold higher affinity toward ARO dioxygenase compared to 2-AP (Fig. 5A, right panel), and its oxidation product, 2-hydroxymuconic semialdehyde, inhibited the enzymatic activity of ARO dioxygenase above a concentration of 50 µM catechol (when more than 300 mol of catechol was oxidized per mole of enzyme) (Fig. S6C). These results indicate that ARO dioxygenase is slightly less sensitive to inhibition by catechol as the other 2-AP 1,6-dioxygenases of *Pseudomonas* species, which are shown to be inhibited when more than 200 mol of catechol was transformed per mole of enzyme (5, 7, 8).

### Functional characterization of the ARO AldDh

To characterize the ability of ARO AldDh to function downstream of the ARO dioxygenase, we attempted to recombinantly express ARO AldDh from *Micromonospora rosaria*. Initially, the expression of an N-terminally His-tagged version of ARO AldDh in *E. coli* resulted in insoluble protein. Neither changing the linker length between the His-tag and the enzyme, nor adding a SUMO domain to its N-terminus, nor fusing the His-tag to the C-terminus, improved its solubility. Expressing the protein without an affinity tag, however, resulted in ~20% soluble protein (Fig. S7A). SDS-PAGE analysis demonstrates our ability to enrich a protein corresponding to a molecular mass of 52 kDa using ammonium sulfate precipitation followed by hydrophobic interaction chromatography (HIC) (Fig. S7B). To verify whether the enriched protein was indeed the ARO AldDh monomer, we tested various ammonium sulfate precipitation fractions and HIC elution fractions for the ability to convert 2-aminomuconate 6-semialdeyde (2-AMS) to 2-aminomuconate *in vitro* (Fig. 5B; Fig. S7C). Since 2-AMS is a highly unstable substrate, the activity of ARO AldDh was detected using a coupled enzyme assay that employed its putative upstream partner, ARO dioxygenase, to generate 2-AMS *in situ*. ARO dioxygenase transforms 2-AP (λ_max_ at 282 nm) to 2-AMS (λ_max_ at 380 nm). 2-AMS will then spontaneously cyclize to picolinic acid (λ_max_ at 263 nm) (Fig. 5B curves 6 and 7, and Fig. 1). However, the addition of 20 µg of the putative AldDh fraction prevents formation of picolinic acid and results in an absorption peak at 326 nm, consistent with the formation of the expected product of the coupled reaction, 2-aminomuconate (Fig. 5B curves 2 and 3). Analyzing the oligomeric state of ARO AldDh by size exclusion chromatography revealed that the enzyme elutes at 13.1 mL, which corresponds to about 150 kDa, suggesting that the active ARO AldDh is a trimer (Fig. S8). This is in contrast with literature where it has been shown to be a homo tetramer (35, 36). However, it is possible that a compact tetramer appears smaller than the size standards from our calibration. Taken together our results establish that the ARO dioxygenase and ARO AldDh work in concert to catalyze the first two steps in the degradation of 2-AP to prevent wasteful intermediates from forming and are likely encapsulated together.

## DISCUSSION

Here, we provide an initial characterization of the ARO BMC encoded by a gene cluster found exclusively in the phylum Actinobacteria using bioinformatics, phylogenetics, structural, functional, and physiological analyses. Studies in other heterotrophs have shown that the 2-AP *meta-*cleavage pathway, which converts 2-AP into pyruvate and acetyl-CoA, requires eight enzymes (Fig. 1). Although we were able to find ARO BMC-encoding species that encode for the full pathway in a single locus, such as in the case of *Micromonospora* and *Verrucosispora*, we also found many species where the main ARO loci were not complete (Fig. 2). In these species, we have identified co-occurrences between the shell proteins and the enzymes involved in only the early stages of the pathway (e.g., ARO dioxygenase, ARO AldDh, 2-aminomuconate deaminase, or 2-hydroxymuconate tautomerase); such conserved patterns of co-occurrence are useful for predicting encapsulation across BMC types (37). In contrast, the genes that encode for the enzymes of the later steps of the 2-AP pathway were less likely to be part of the main operon but usually found in satellite loci downstream of an IclR-type transcription regulator that is also found in the ARO locus (Fig. 2A; Fig. S2). IclR transcription regulators act as repressors and bind DNA in the absence of an effector. They have been shown to be involved in the regulation of aromatic compound degradation pathways such as p-hydroxybenzoate in *Pseudomonas putida* (38) and protocatechuate in *Acinetobacter* sp. ADP1 (39) review in reference (40). It is not yet clear whether these remote IcIR-type regulator-associated genes are part of the ARO enzymatic core and whether their expression is co-regulated with the primary ARO locus. There are differences in the amino acid sequences of the IclR effector binding domain from both main and satellite loci (Fig. S9A), suggesting that they may be regulated by different effectors. Consistent with such a hypothesis is that the IclR domain associated with satellite loci containing the tautomerase is on a distinct branch of a phylogenetic tree (Fig. S8B).

In addition to the suggestion that the ARO BMC degrades 2-AP (15), we have identified ARO loci that encode the 2-hydroxymuconate tautomerase of the catechol *meta*-cleavage pathway (Fig. 1; Fig. 2A). Both 2-AP and catechol degradation BMC loci are very similar and only differ in the presence of the gene coding for one pathway-specific protein, 2-aminomuconate deaminase, or 2-hydroxymuconate tautomerase. Indeed, our enzymatic analyses indicate that the ARO dioxygenase is a bifunctional enzyme that can act on both 2-AP and catechol (Fig. 5; Fig. S6). Although we observed an inhibition effect of catechol at high concentration (Fig. S6C, >50 µM) *in vitro* as previously observed (5 - 7), this is likely due to product inhibition and will not affect activity *in vivo*, especially when ARO dioxygenase and ARO AldDh are both confined in the BMC. Additionally, the 2-aminomuconate 6-semialdehyde dehydrogenase is generally also able to process substrates from both pathways (10, 35). A homology model of the ARO AldDh based on the homologous structure of 2-aminomuconate-6-semialdehyde dehydrogenase from *Pseudomonas fluorescens*, which was shown to work on both 2-hydroxy and 2-aminomuconate-6-semialdehyde (36), shows that the active sites are very similar with only a single conservative (Tyr, *P. fluorescens* to Phe [ARO]) substitution (Fig. S3B), indicating that the ARO AldDh is likely to be able to process substrates from both pathways. In addition, our phylogenetic analysis shows that ARO AldDh can be found in the same clade with other non-BMC-associated nitrobenzene-degrading AldDhs (Fig. 3B). This suggests that, depending on the species, it could be that the ARO BMCs evolved to degrade either 2-AP or catechol-derived substances, products of as-yet-to-be-characterized upper pathways.

Although the identity of the physiological substrate of the ARO BMC in *M. rosaria* is unknown, both the structural resemblance of ARO dioxygenase from *M. rosaria* to a known 2-AP 1,6-dioxygenase and its clear preference to cleave a benzene ring with an amino group rather than hydroxyl or chlorine groups (Fig. 5) strongly suggest that the physiological substrate is being funneled into 2-AP by an unknown upper pathway. However, unlike some nitrobenzene- and chlorobenzene-degrading bacteria that were shown to grow on 2-AP (22, 30, 41), *M. rosaria* cells were not able to utilize 2-AP as a sole carbon and nitrogen source (Fig. S4A) and were only able to grow in the presence of low 2-AP concentrations on rich media (Fig. S4B). Furthermore, high 2-AP concentrations (>100 µM) resulted in the toxicity and slowing of cell growth on a rich media (Fig. S4B), which might be the result of a metabolic misrouting or production of a toxic intermediate from 2-AP. The inability to tolerate high concentrations of 2-AP, despite the presence of an active 2-AP degradation pathway, as demonstrated in our *in vitro* activity assays (Fig. 5), indicates that the expression of the ARO enzymatic core is most likely tightly regulated by the upstream molecule that binds the IclR-type transcription regulator and prevents it from binding to the DNA. Indeed, many studies of known metabolosomes have shown that the loci encoding for their shell proteins and enzymatic core are only expressed in response to environmental cues, or in response to a metabolite, such as the substrate of the metabolosome, given the relatively expensive metabolic cost of expressing BMCs (42 - 46). Interestingly, there are several nitrobenzene-degrading *Pseudomonas* strains that cannot grow on 2-AP despite the presence of the *amn* gene cluster in their genome (47, 48) or that their ability to grow on 2-AP was only observed when the cells were previously induced by nitrobenzene (34). In these cases, it was demonstrated that nitrobenzene (e.g., an upstream molecule) was the sole inducer of the catabolic pathway, and none of the downstream products, including 2-AP, were able to induce the expression of the 2-AP degradation enzymes. Therefore, the inability of 2-AP to induce the expression of the ARO BMC locus may indicate that if 2-AP is not the physiological substrate of the ARO BMC, its substrate(s) are aromatic metabolites that structurally resemble 2-AP or catechol.

Among the enzymes involved in the 2-AP pathway, we were especially interested in the ARO dioxygenase, which we posited to be the ARO BMC signature enzyme, and the ARO AldDh which is central to the reaction pathway and involved in the detoxification of the aldehyde intermediate (16). Both the confinement of ARO dioxygenase to ARO loci in Actinobacteria (Fig. 3A) and the lack of density for the N-terminus of the beta subunit, typical for encapsulation peptides (Fig. 4) (31 - 33), support the role of the ARO dioxygenase as an encapsulated enzyme, which has diverged from cytosolically localized counterparts in terms of both its structure (potential EP and shielded catalytic site) and the enzyme’s substrate specificity (6, 7, 30). How the ARO AldDh would get encapsulated, however, is still uncertain. The majority of BMCs found in heterotrophs encapsulate an aldehyde dehydrogenase sequestered in the shell with a signature enzyme that generates an aldehyde (16, 18). However, in contrast to all BMC-associated aldehyde dehydrogenases, the ARO AldDh does not contain an encapsulation peptide (EP). Nevertheless, its co-occurrence with ARO dioxygenase and the shell proteins in all the ARO loci, as well as its ability to work in concert with the ARO dioxygenase *in vitro* to prevent accumulation of dead-end products (Fig. 5B), suggests that it is likely encapsulated together with ARO dioxygenase. Because EPs facilitate interactions with the shell (23, 49, 50), we posit its encapsulation might be mediated differently than in other known metabolosomes (17). This is consistent with the significant distance of the ARO shell proteins from any other BMC type on a phylogenetic tree (15).

**Fig 6:**
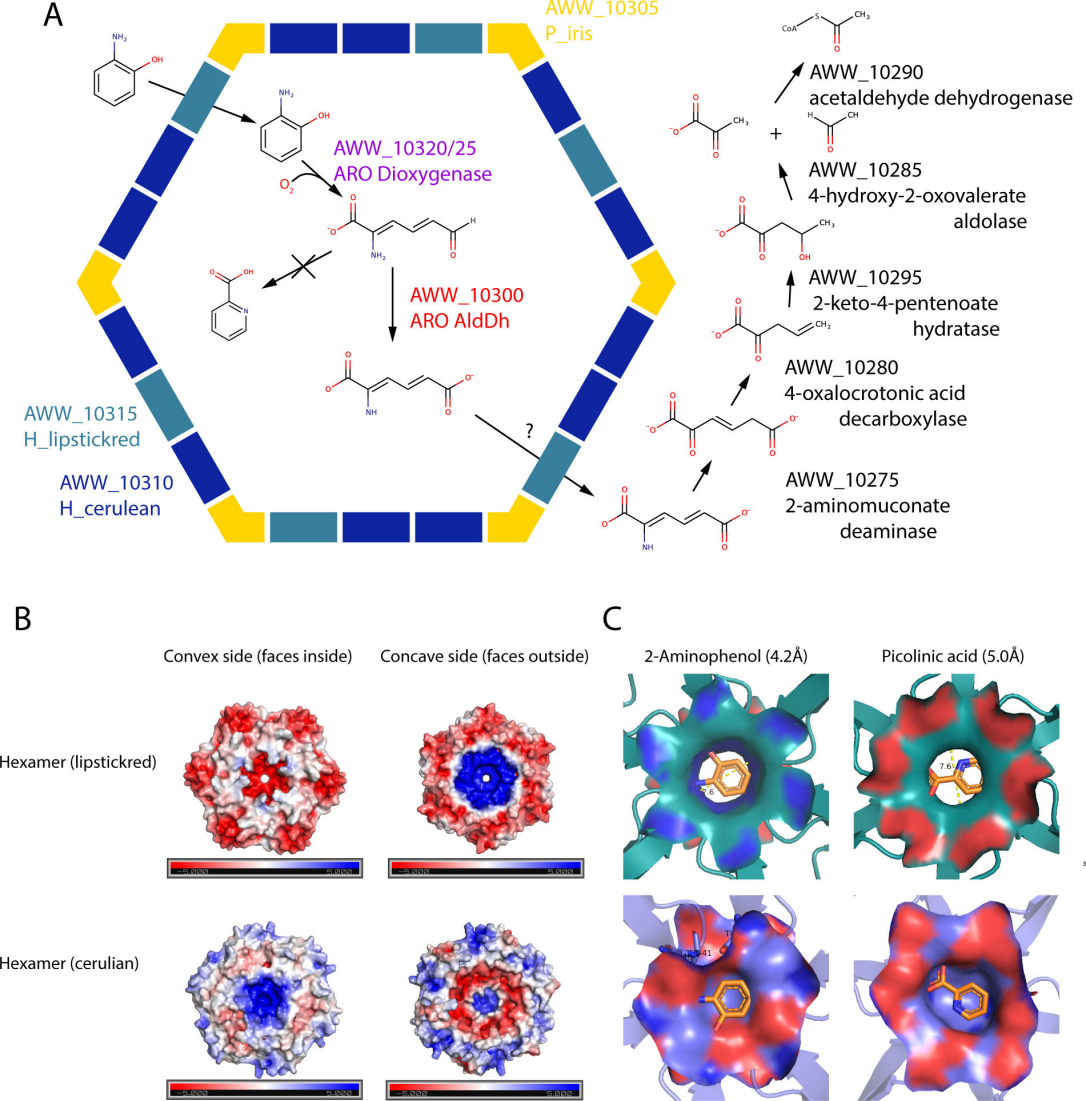
**(A**) Proposed model of the 2-aminophenol pathway in a *M. rosaria* with an ARO BMC shell schematic. Our schematic proposes encapsulation of ARO dioxygenase and ARO AldDh, but location of further downstream enzymes in the pathway remains to be determined. Gene products are given by their locus tags in IMG and their annotated function. (**B**) Electrostatic surface analyses of ARO lipstickred (upper panel) and cerulean (lower panel) hexamer oligomers. Both the concave and convex sides of the model of each shell protein oligomer are shown. Positive surface potentials (blue) and negative surface potentials (red) ranging from 5 to −5 kT/e are indicated. (**C**) Modeling of 2-AP (left panel) or picolinic acid (right panel) in the pore region of ARO H_lipstickred (upper panel) or H_cerulean (lower panel) hexamers. The left and right panels show concave and convex sides, respectively.

It is an intriguing observation to find ARO dioxygenase and ARO AldDh confined to a BMC exclusively in the Actinobacteria. Typically, enzymes are encapsulated into BMCs to improve enzyme kinetics through scaffolding to sequester enzymes with substrates, to confine toxic or volatile intermediates (such as aldehydes), or to protect oxygen-sensitive enzymes (15, 16, 18, 19, 51). Our functional analysis showed that the ARO dioxygenase is not sensitive to oxygen; however, its catalytic efficiency was one magnitude lower than reported previously for 2-AP 1,6-dioxygenase from nitrobenzene- and chloronitrobenzene-degrading bacteria (Fig. 5) (5 - 25 - 25). Therefore, encapsulating the ARO dioxygenase could be important for improving catalytic efficiency. Another possibility is that encapsulating ARO dioxygenase together with ARO AldDh prevents the formation of toxic or dead-end products. Studies have shown that the 2-AP *meta*-cleavage pathway generates toxic aldehydes and dead-end products (34, 52, 53). Indeed, to date, only nitrobenzene- and chlorobenzene-degrading bacteria were shown to utilize this pathway, possibly due to its disadvantages. We were able to detect the formation of picolinic acid in our functional assay, following the oxidation of either 2-AP or 2A5CP in the ARO dioxygenase functional assay (Fig. S6) or when ARO AldDh or NAD^+^ was excluded from the coupled enzyme assay (Fig. 5B, curves 4 and 7). It is reasonable to assume that a co-localization of the ARO dioxygenase and the ARO AldDh is required for the rapid conversion of the intermediate to prevent the formation of picolinic acid, ensuring the continuation of the pathway to the formation of pyruvate. Therefore, based on our results, we suggest that the ARO dioxygenase converts the 2-AP-derived substance into an aldehyde within the BMC lumen, which is then detoxified by the encapsulated ARO AldDh, which converts it into its acid form (Fig. 6A). The confinement of the 2-AP *meta*-cleavage pathway within shells not only ensures a quick conversion of the intermediate but also offers the bacteria a sophisticated way to overcome the 2-AP pathway disadvantage and enable the utilization of metabolic pathways that otherwise were not accessible due to toxicity. Whether further downstream enzymes of the pathway are localized in the shell has yet to be determined.

Although it is not yet firmly established, the ARO BMC could be the first BMC that is involved in the degradation of aromatic substrates. While xanthine has been proposed as the substrate of a functionally uncharacterized BMC (54), we support our hypothesis for a degradation pathway for 2-AP or catechol with bioinformatic analysis, structural, and enzymatic characterization. Typically, in experimentally characterized BMCs, the size and charge of the pores of at least one of the facet shell proteins can be identified as the likely conduit for the signature enzyme substrate. The ARO BMC is unusual for its very simple shell composition of only two BMC-H and a BMC-P that are deeply divergent of other metabolosome shells (15, 21). Based on homology modeling and electrostatic surface analyses (Fig. 6B and C), the two hexamers differ both in size and in net charge in the area around the pores. While the H_lipstickred is characterized by a wide pore (7.6 Å) and strong negative and positive charge on the convex and concave sides, respectively, the H_cerulean has a relatively small pore (5.6 Å) and a relatively non-polar pore environment (Fig. 6B). The slightly non-polar character of 2-AP suggests that the aromatic molecule is not being repelled by either pore, and it is most probably the size and not the charge of the pore that determines through which hexamer the 2-AP or catechol will cross into the shells. The H_lipstickred pore is wide enough to allow its passage (Fig. 6C, left panel). Accordingly, we suggest that H_lipstickred is likely the hexamer that allows the movement of 2-AP through the pore, while H_cerulean may play a role in releasing the converted intermediates or carboxyl and amine groups that are formed during the pathway. Interestingly, our homology modeling analysis demonstrates that in the case that picolinic acid does form, it would likely not traverse the pores of either hexamer because of size and/or charge restrictions (Fig. 6C, right panel).

Here, we show that ARO BMCs likely degrade either 2-AP or catechol, but the nature of the upstream substrate that is converted into these central aromatic intermediates is unknown. In contrast to the nitrobenzene-degrading bacteria that were mostly found in aromatic pollutants-contaminated areas, which usually contain a high concentration of the degraded pollutant, the ARO BMC loci-containing species are found in natural environments such as soil and aquatic sediments, where there is no evidence for the presence of aromatic pollutants. The existence of a dedicated microcompartment for the degradation of 2-AP or catechol in members of Actinobacteria is more likely to be due to the presence of a naturally occurring upstream molecule of 2-AP rather than industrial pollutants. Because we could not identify enzymes for an upper pathway in the ARO BMC locus, those enzymes are likely cytosolic. Known metabolosomes, such as the propanediol- and ethanolamine-degrading BMCs [PDU and EUT, respectively (55, 56)], have shown to only be induced in the presence of the initial substrate. Some more recently characterized metabolosomes, such as the Planctomycete and Verrucomicrobia microcompartment (PVM) BMC (42) or the taurine-degrading BMC (57), however, are induced by molecules upstream of the reaction catalyzed inside the BMC. For those two examples, those enzymes are cytosolic and not found associated with the BMC locus. The only possible candidate for an upper pathway enzyme in the ARO BMC locus is the HyuB gene that is located upstream of the ring-opening enzyme, which its partner, HyuA, if not found within the locus, was usually found in very close proximity to it (Fig. 2; Fig. S2). HyuB encodes for subunit B of a methylhydantoinase with unknown function. BLAST search of HyuB from *Micromonospora rosaria* against known structures reveals similarity of 20%–30% to acetophenone carboxylase (Apc) β subunit, which is responsible for the carboxylation of ketone acetophenone to benzoylacetate to promote its degradation (58). Although the role of the HyuAB enzyme is not clear, it is possible that it functions upstream of the *meta*-cleavage pathway and decarboxylates an aromatic molecule to convert it to 2-AP or catechol.

In summary, our study provides an initial functional characterization and lays the foundation for future studies for understanding the physiological role of the ARO BMC. The existence of a dedicated microcompartment for the degradation of 2-AP or catechol in members of Actinobacteria suggests that the BMC shell enables an otherwise inaccessible catabolic pathway. In addition, it further exemplifies the metabolic flexibility that this phylum is known for, which allows its members to occupy different niches. The fact that several of the ARO BMC-encoding species encode more than one type of BMC, some of an unknown function (15), likely further contributes to this versatility.

## MATERIALS AND METHODS

### Bacterial strains and culture conditions

*Micromonospora rosaria* DSM 803 was cultivated in ISP2 medium (0.4% yeast extract, 1% malt extract, 0.4% glucose, and 10 mM CaC0_3_ pH 7.2 that was adjusted by NaOH) at 28°C on a rotary shaker (180 rpm). To determine the ability of *M. rosaria* to utilize 2-AP as carbon and nitrogen source, we performed an assay as previously described (59). Cells were grown until mid-log phase in ISP2 rich media and then diluted 1:20 to a minimal media [10 mM KH_2_PO_4_, 5 mM MgSO_4_, 10 µM FeSO_4_, 10 µM ZnSO_4_, 10 µM CuSO_4_, 1 µM MnSO_4_, and 10 mM CaCO_3_, supplemented with cysteine (0.1 mg/L) and vitamins] with different concentrations of 2-AP. One hundred millimeters of glucose and 25 mM of NH_4_NO_3_ were used as carbon and nitrogen source, respectively. Growth was determined by spreading 50 µL on ISP2 plates supplemented with 0.2% CaCO_3_ and incubated at 28°C until colonies appeared.

### Bioinformatic analysis

The ARO locus database was extracted from the work of Sutter et al. (15) and was updated with a BLAST search of H_lipstickred from the *Micromonospora rosaria* against all isolated genomes in IMG (img.jgi.doe.gov). We defined ARO BMC satellite loci as those that meet three criteria: the main ARO locus contains an insufficient enzymatic core required for the whole pathway, more than one gene of the 2-aminophenol pathway is found adjacent to each other, and the gene for an IclR-type regulator is found next to them. For the ARO dioxygenase beta subunit N-terminal region analysis (Fig. S3A), protein sequences were obtained using standard cutoff NCBI BLAST search with the *Micromonospora rosaria* beta subunits (see Supplementary Data Set 1). Multiple-sequence alignments were performed with the MAFFT server (60) using G-INS-I parameters, and alignments were visualized using the JalView program (61). Evolutionary analyses were conducted in the MEGA (version 11) program (62) with 100 bootstraps.

For the ARO dioxygenase tree (Fig. 3A), protein sequences were obtained using a 1E-25 cutoff NCBI BLAST search with the *Micromonospora rosaria* alpha and beta subunits. Hits were then combined, all known ARO sequences added to recognize them on the tree later, and all duplicates removed. The 870 protein sequences were aligned with ClustalW (63) using standard parameters, trimmed with trimAl (64) using parameters “-gt 0.6 –cons 30 –w 3,” and a tree with 100 bootstraps generated with RaXML (65) using a PROTGAMMAWAG substitution model. For the aldehyde dehydrogenase tree (Fig. 3B), a similar procedure was used, selecting the best 500 Actinomycete hits of the *Micromonospora rosaria* aldehyde dehydrogenase. Homology modeling was performed by using the SWISS-MODEL online tool (66) using the following PDB IDs: 4I25 for 2-aminomuconic semi-aldehyde dehydrogenase, 5DJB for ARO H_lipstickred, and 3SSS for ARO H_cerulean.

### Cloning and heterologous expression of the alpha and beta subunits of ARO dioxygenase from *Micromonospora rosaria*

The *Escherichia coli* codon-optimized genes for the alpha and the beta subunits of 2-aminophenol-1,6-dioxygenase from *Micromonospora rosaria* were synthesized (Integrated DNA technologies, Coralville, IA, USA ) and cloned into pACYCDuet and pCDFDuet1 expression plasmids, using NdeI and XhoI (Fw: ATCATATGGCAGATCGGACCGGGAT; Rev: ATCTCGAGTTAAGGGTGGTCAGGAAGATTCCAG) or NdeI and BglII (Fw: ATCATATGTCCCGCGTATTCCGGG; Rev: ATGGATCCTCAGTGGTGATGGTGATGATGactaccacctgatccGCCGTGGTGAGACCCC) restriction sites, respectively. The beta subunit was cloned with a 6xHis affinity tag at the C-terminus that was separated from the protein through a 2xGly_Ser_Ser linker. The resulting plasmids (pLD54 and pLD59 for the alpha and His-tagged beta subunits, respectively) (see Supplementary Data Set 2 for nucleotides [codon-optimized] and amino acid sequences) were transformed into *E. coli* BL21 (DE3) cells and induced with 0.25 mM isopropyl β-D-1-thiogalactopyranoside at mid-exponential phase (OD_600_ nm = 0.6–0.8). The culture induced for 4 hours at 37°C. Cells were harvested by centrifugation and resuspended in buffer containing 0.01 M Tris-HCl pH 8.0, 0.5 M NaCl, 20 mM imidazole, and 5% glycerol (Buffer A) at a density of 0.5 g/mL and stored at −20°C until lysis.

### ARO dioxygenase purification

Cell pellets were thawed in their respective buffer and SigmaFast Protease Inhibitor (Sigma Aldrich, St. Louis, MO, USA), 0.1 mg/mL lysozyme (Sigma Aldrich, St. Louis, MO, USA), and 1 mg/mL DNase (Sigma Aldrich, St. Louis, MO, USA) were added. Cells were incubated on ice for 15 minutes following their lysis using a French Press at >1,000 psi. Crude lysate was clarified by centrifugation at 40,000 × *g* for 45 minutes at 4°C, and supernatant was applied to a 5-mL HisTrap column (GE Healthcare, Chicago, IL, USA) that had been equilibrated with Buffer A. The column was washed with Buffer A with 100 mM imidazole, and the recombinant enzyme was eluted with the same Buffer A with 200 mM imidazole. Fractions containing both subunits of the dioxygenase were pooled and concentrated using an Amicon Ultra-4 Centrifugal Filter Unit (EMD Millipore, Burlington, MA, USA) with a 50-kDa pore size. The sample was then applied to a 120-mL gel filtration column (HiLoad 16/600 Superdex 200 pg, GE Healthcare), equilibrated with 0.01 M Tris-HCl pH 8.0, 0.5 M NaCl, and 5% glycerol, and eluted at 180 mL which is equal to 140 kDa (according to its elution volume of 13.3 mL on analytical Superdex 200 300/10 Gl, GE Healthcare column, Fig. S4C), corresponding to a dimer of heterodimers. The collected fractions were desalted by passage through a PD-10 column (Pharmacia, Mississauga, Ontario, Canada) equilibrated with a buffer containing 10 mM Tris-HCl pH 8.0 + 5% glycerol.

### Cloning and heterologous expression of ARO AldDh from *Micromonospora rosaria*

The *E. coli* codon optimized gene for ARO AldDh from *M. rosaria* was synthesized (Integrated DNA technologies) and cloned into pBbE2k expression plasmids, using NdeI and BamHI restriction sites (primers: Fwd: TACATATGACTGGCCTTTGGTCTCCTACATTAC; Rev: TTGGATCCTCAGCTCAAATCCACG). The enzyme was cloned without affinity tag. The resulting plasmid (pLD69) (see Supplementary Data Set 2 for nucleotides [codon-optimized] and amino acid sequences) was transformed into *E. coli* BL21 (DE3) cells and induced with 100 ng anhydrotetracycline (aTc) at OD_600_ = 0.6–0.8. The culture was induced for 4 hours at 37°C. Cells were harvested by centrifugation and resuspended in buffer containing 50 mM Tris-HCl pH 8.0, 0.2 M NaCl, and 0.2 mM NAD^+^ (Buffer B) at a density of 0.5 g/mL and stored at −20 °C until lysis.

### ARO AldDh purification

Cell pellets were thawed in their respective buffer, and SigmaFast Protease Inhibitor (Sigma Aldrich, St. Louis, MO, USA), 0.1 mg/mL lysozyme (Sigma Aldrich, St. Louis, MO, USA), and 1 mg/mL DNase (Sigma Aldrich, St. Louis, MO, USA) were added. Cells were incubated on ice for 15 minutes following their lysis using a French Press at >1,000 psi. Crude lysate was clarified by centrifugation at 40,000 × *g* for 45 minutes at 4°C, and supernatant volume was increased to 100 mL using Buffer B. Ammonium sulfate was added in 5% intervals (30 minutes incubation at 4°C each) followed by centrifugation at 15,000 × *g* for 15 minutes. The pellet from the 40% and 45% ammonium sulfate precipitation was resuspended with 50 mM Tris pH 8.0, 1 M ammonium sulfate, 0.2 mM NAD^+^, and 1 mM DTT (Buffer C) and applied to a 5-mL HiTrap Phenyl HP column (GE Healthcare) that had been equilibrated with Buffer C. The column was washed with Buffer C, and the recombinant enzyme was eluted at 80% Buffer D (50 mM Tris-HCl pH 8.0, 0.2 mM NAD^+^, and 1 mM DTT). The sample was then applied to a 120-mL gel filtration column (HiLoad 26/600 Superdex 200 pg, GE Healthcare), equilibrated with 0.01 M Tris-HCl pH 8.0, 0.3 M NaCl, 1 mM DTT, and 0.2 mM NAD^+^, and eluted at 180 mL which is equal to 152 kDa (according to its elution volume of 13.1 mL on analytical Superdex 200 300/10 GL, GE Healthcare column, Fig. S4C), corresponding to a dimer of heterodimers. Fractions were further pooled and concentrated using a Amicon Ultra-4 Centrifugal Filter Unit (EMD Millipore) with a 50-kDa molecular weight cutoff, followed by desalting using a PD-10 column (Pharmacia) equilibrated with buffer containing 10 mM Tris-HCl pH 8.0, 0.5 M NaCl, 0.2 mM NAD^+^, 1 mM DTT, and 5% glycerol (Buffer E). For analytical size exclusion analysis, the sample was applied on analytical Superdex 200 10/300 GL, GE Healthcare column, equilibrated with Buffer E, and eluted at 13.1 mL, corresponding to 152 kDa as calculated from molecular weight standards (Fig. S8A).

Protein concentrations were quantified via the Pierce BCA Protein Assay Kit using bovine serum albumin in comparable buffers for constructing standard curves.

### Enzyme kinetics measurements

Catalytic kinetics measurements of 2-aminophenol-1,6-dioxygenase on 2-aminophenol, 2-amino-5-chlorophenol, and catechol were performed at room temperature by monitoring the increase in absorbance at 380 nm for 2-aminophenol, 395 nm 2-amino-5-chlorophenol (due to the formation of 2-aminomuconic acid semialdehyde and 2-amino-5-chloromuconate 6-semialdehyde), and at 375 nm for catechol (due to the formation of 2-hydroxymuconic acid semialdehyde). The reaction mixture (1 mL) contained 10 mM sodium phosphate buffer (pH 8.0) and 27 µg enzyme, and the reaction was started by adding the different substrates. The *Km* and *Vmax* values were calculated from Lineweaver–Burke plots using the following molar extinction coefficients: 15,100 M^−1^ cm^−1^ for 2-aminophenol, 21,000 M^−1^ cm^−1^ for 2-amino-5-chlorophenol (8), and 33,000 M^−1^ cm^−1^ for catechol (12). The results were obtained from three independent replicate experiments.

The activity of ARO AldDh was determined by monitoring the conversion of 2-AMS (λ_max_ at 380 nm) into 2-aminomuconate (λ _max_ at 326 nm) in a coupled enzyme assay that employed ARO dioxygenase, to generate 2-AMS *in situ*. The reaction mixture contained 100 mM potassium phosphate (pH 7.5), 0.5 µM 2-AP, 27 µg of purified dioxygenase per milliliters, 20 µg of purified ARO AldDh, 20 µM NAD^+^. The reaction was started by adding 2-AP. The results were obtained from three independent replicate experiments.

### Structure determination

Crystals of the ARO dioxygenase were obtained by vapor diffusion in sitting drop experiments at room temperature. Two  microliters of protein solution (4.2  mg/mL in 10  mM Tris pH 8.0, 5% glycerol) were mixed with 2  µL of reservoir solution containing 0.1 M Tris pH 8.0, 0.5  M ammonium, and 28% PEG 8000. Crystals were stabilized by adding 4 µL 50% PEG 400 to the drop, mounted on a nylon loop and frozen in liquid nitrogen. X-ray diffraction data were collected at beam line 5.0.1 of the Advanced Light Source at Lawrence Berkeley National Lab. Diffraction data were integrated and scaled with XDS (67) and merged with aimless (68). Electron density was obtained using molecular replacement with phenix.phaser (69) with a homology model based on PDB ID 3VSG. Four dimers of alpha/beta heterodimers were located in the asymmetric unit, and models were built using coot (70) alternating with refinement with phenix.refine (71). Statistics for diffraction data collection, structure determination, and refinement are found in Table S1. The coordinates have been deposited at the PDB with accession code 7TXY.

## Data Availability

The coordinates for the crystal structure of the 2-aminophenol 1,6-dioxygenase from the ARO bacterial microcompartment of *Micromonospora rosaria* have been deposited at the PDB with accession code 7TXY.

## SUPPLEMENTAL MATERIAL

Data Set 1. mbio.01216-23-s0001.xlsx NCBI BLAST ARO 2-aminophenol 1,6-dioxygenase all isolate.

Data Set 2. mbio.01216-23-s0002.xlsx Codon optimized gene sequence.

Fig. S1 to S9 and Table S1 mbio.01216-23-s0003.docx Fig. S1 to S9 and Table S1 with legends for figures, table, and Data Sets S1 and S2.

ASM does not own the copyrights to Supplemental Material that may be linked to, or accessed through, an article. The authors have granted ASM a non-exclusive, world-wide license to publish the Supplemental Material files. Please contact the corresponding author directly for reuse.
