# Supplementary material for: Characterization of a novel aromatic substrate-processing microcompartment in Actinobacteria
Source: mBio. 2023 Jul 18;14(4):e01216-23. doi: 10.1128/mbio.01216-23 (PMC10470539; doi:10.1128/mbio.01216-23)
Supplement: Fig. S1 to S9 and Table S1 — with legends for figures, table, and Data Sets S1 and S2. [file mbio.01216-23-s0003.docx]

**Supplementary Information for**

Characterization of a novel aromatic substrate processing microcompartment in Actinobacteria

Lior Doron, Markus Sutter, and Cheryl A. Kerfeld

Cheryl A. Kerfeld

**Email:**  ckerfeld@lbl.gov

**This PDF file includes:**

Figures S1 to S9

Tables S1

**Supplementary Figures**

**Figure S1.**


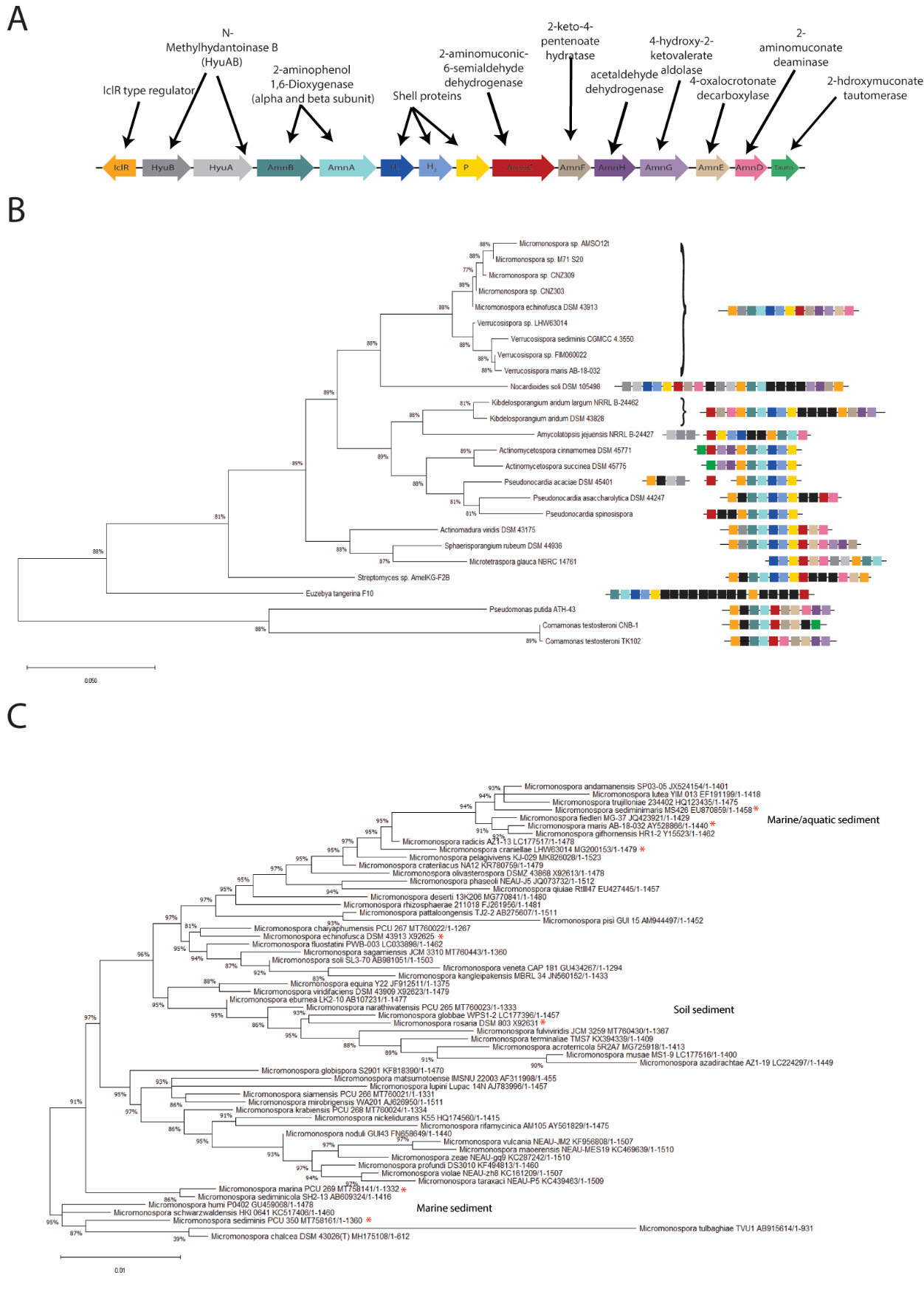


**Figure S2.**

**
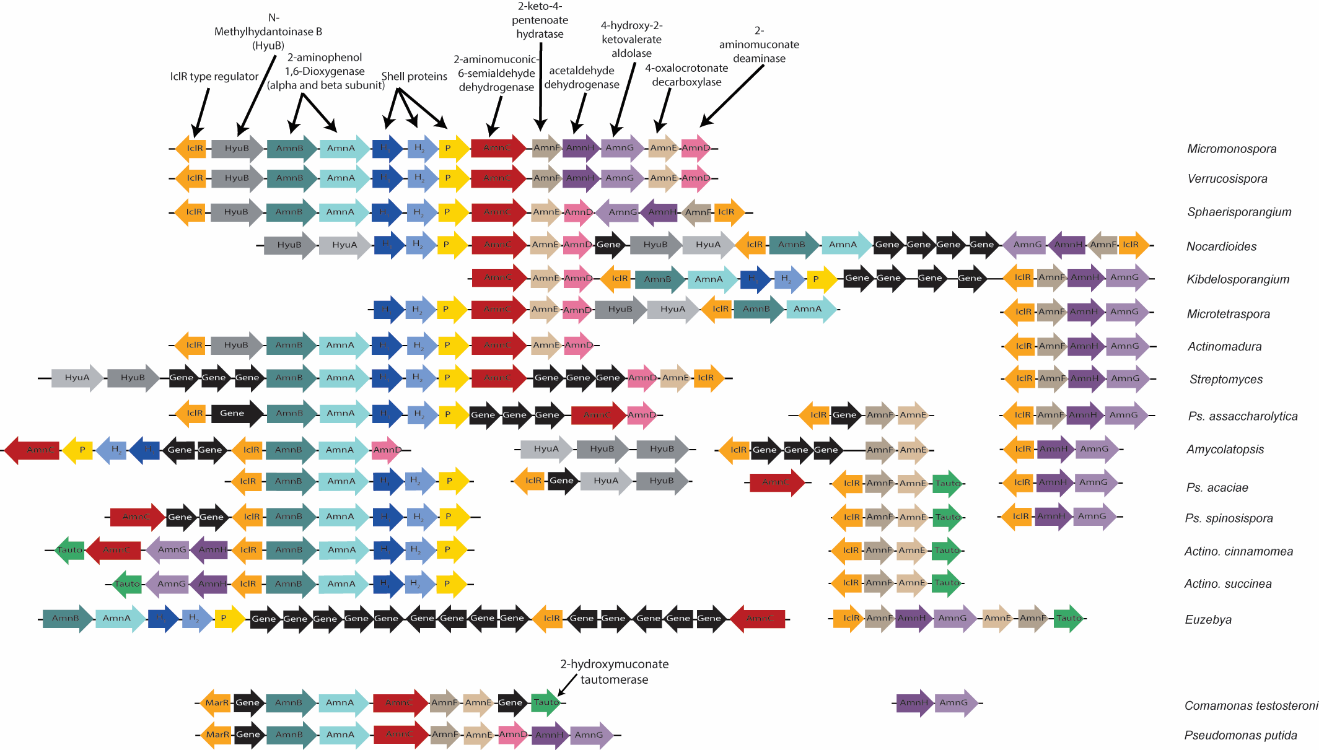
**

**Figure S3.**


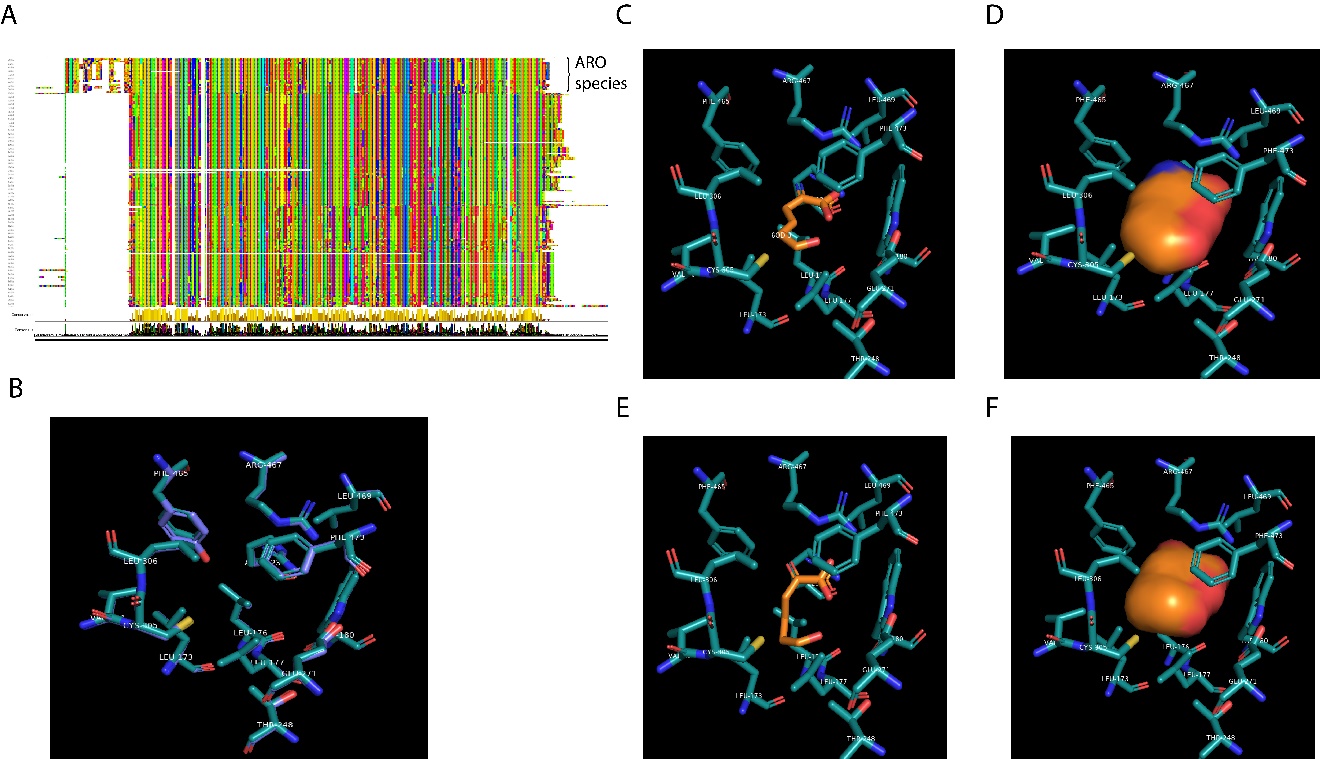


**Figure S4.**

**
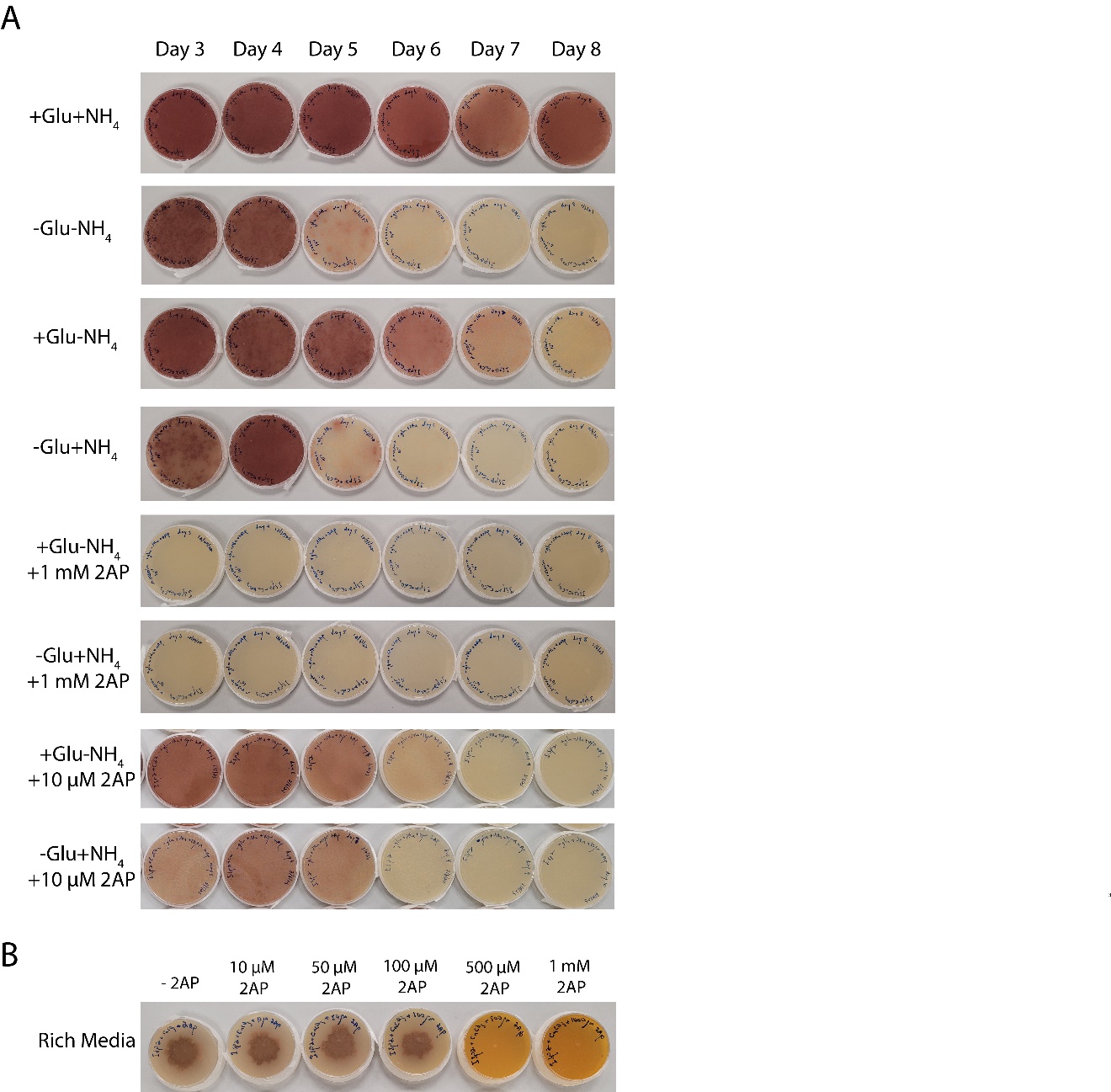
**

**Figure S5.**


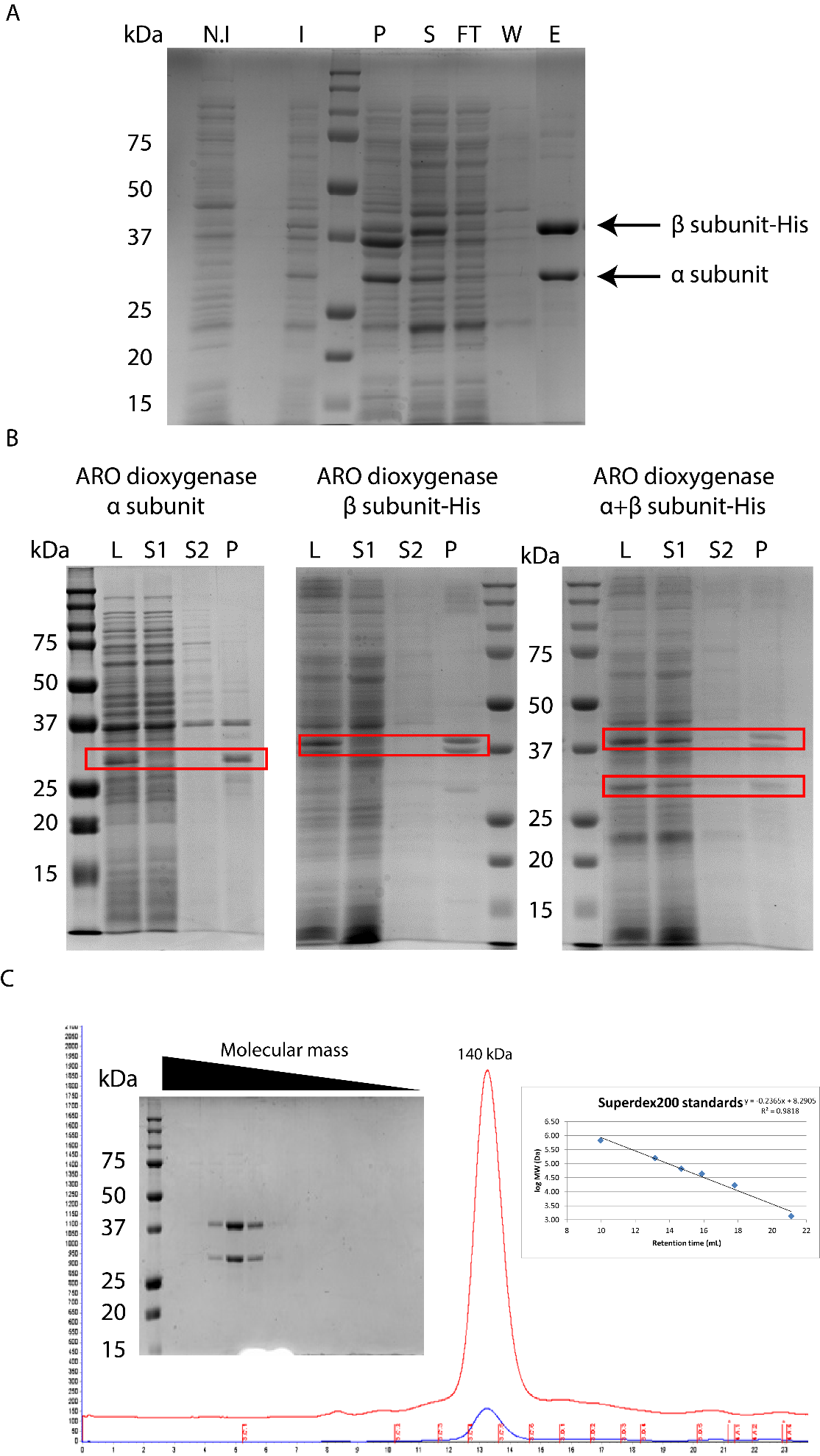


**Figure S6.**


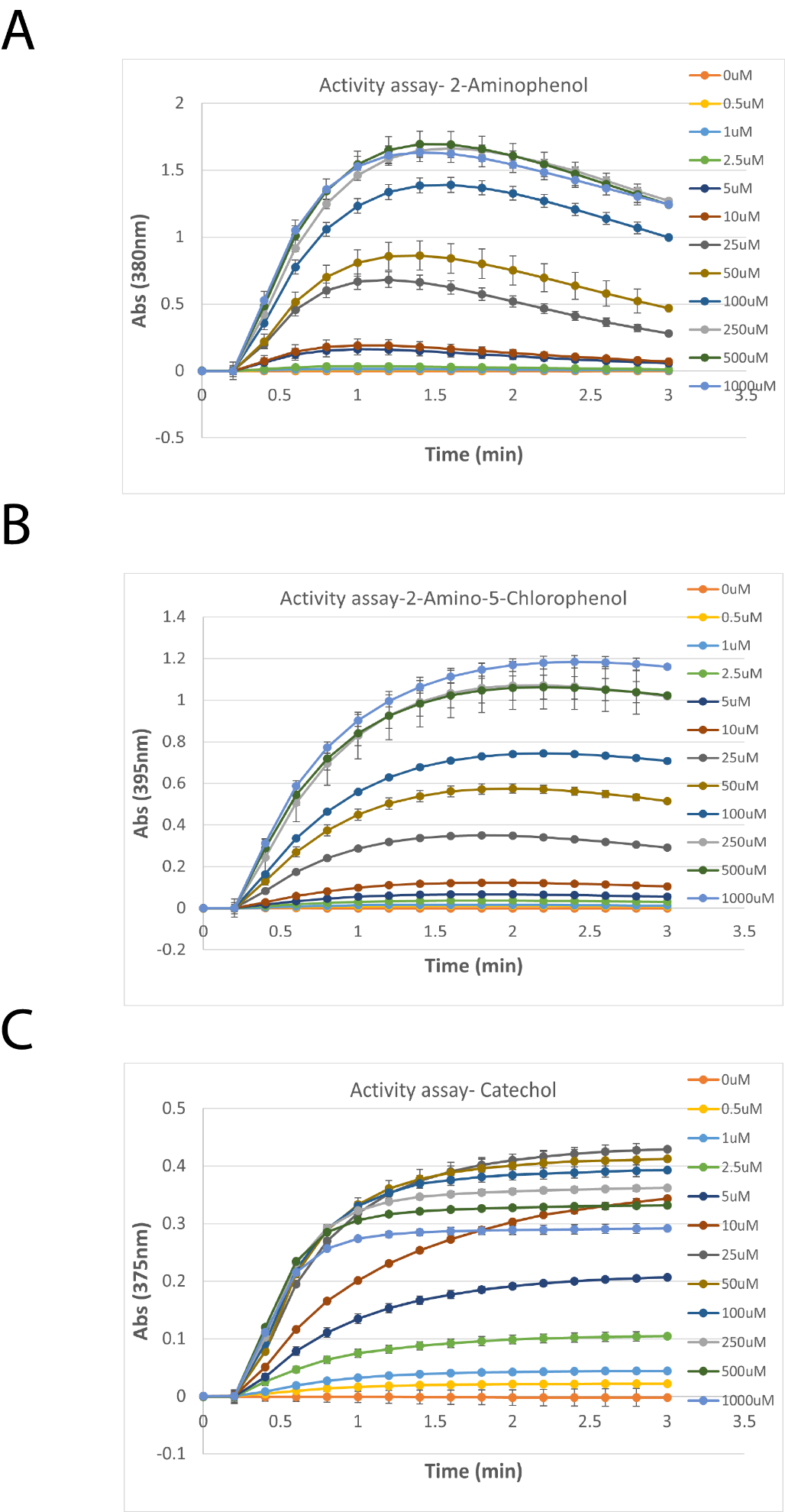


**Figure S7.**


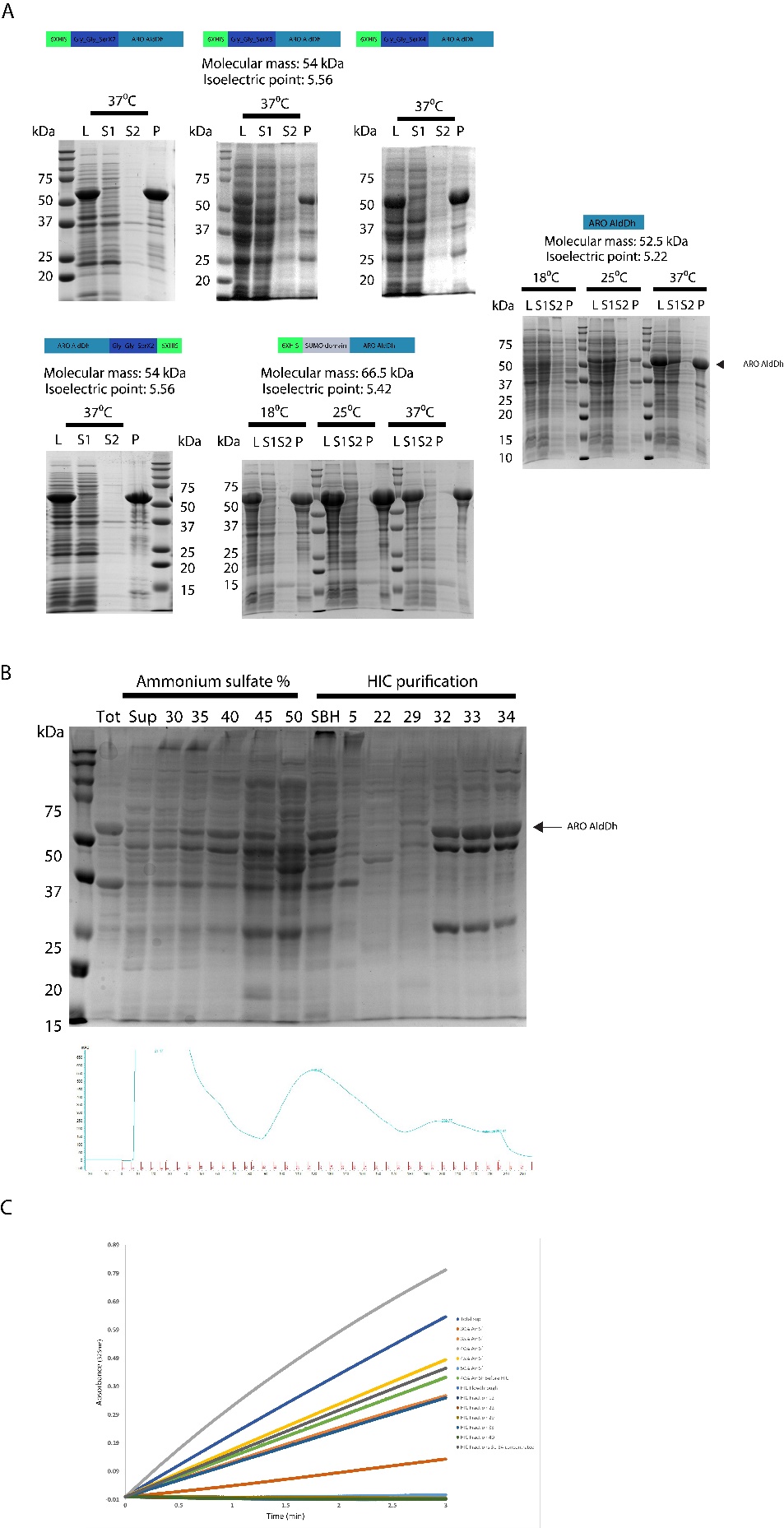


**Figure S8.**


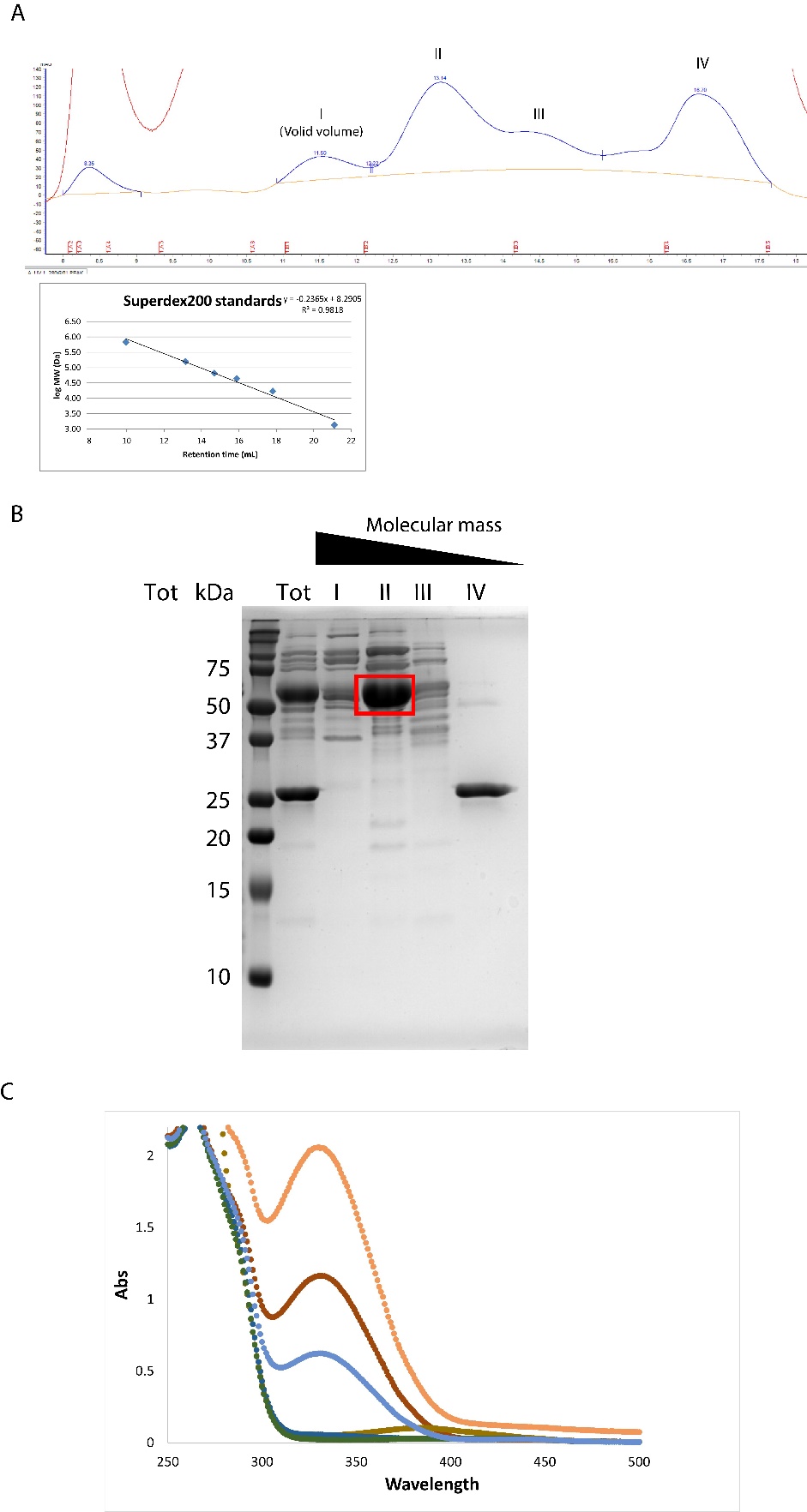


**Figure S9.**

**
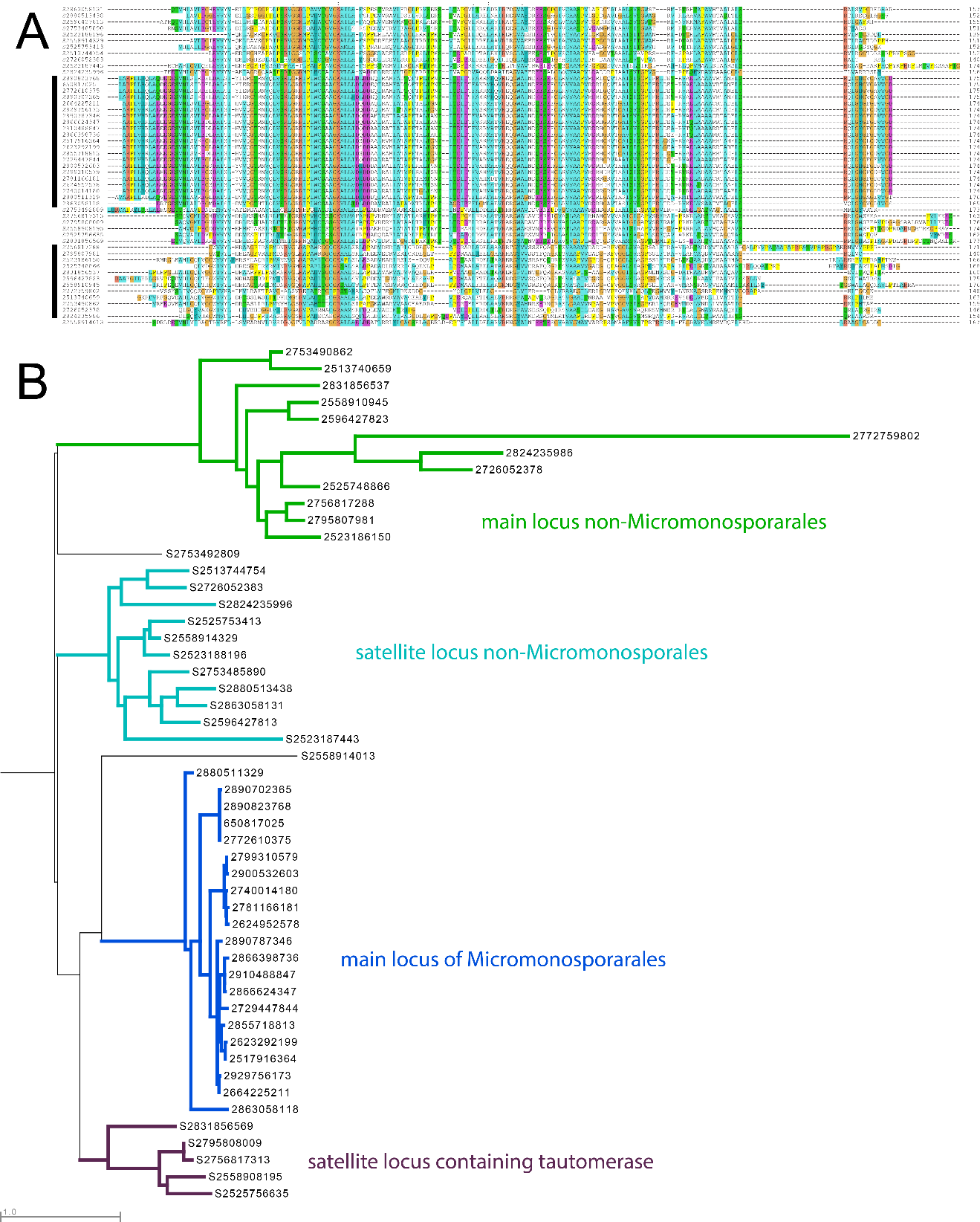
**

Table S1. Data collection and refinement statistics of the *Micromonospora rosaria* 2-aminophenol 1,6-dioxygenase

| **Data collection** | **ARO dioxygenase** |
| --- | --- |
| Wavelength (Å) | 0.9774 |
| Resolution range (Å) | 48.5 - 1.75 (1.78 - 1.75) |
| Space group | P 1 |
| Unit cell dimensions | 68.8 83.9 110.3 Å |
|  | 85.6, 73.1, 89.3° |
| Total reflections | 813,257 |
| Unique reflections | 230,720 (11,288) |
| Multiplicity | 3.5 (3.6) |
| Completeness (%) | 97.3 (95.7) |
| Mean I/sigma(I) | 15.1 (0.8) |
| R-merge | 0.036 (1.173) |
| R-meas | 0.051 (1.659) |
| CC^1/2^ | 0.998 (0.421) |
| **Refinement** |  |
| Resolution range (Å) | 43.6 - 1.75 (1.81 - 1.75) |
| Number of reflections | 230627 (22654) |
| Number of reflections used for R-free | 1984 (200) |
| R-work (%) | 19.4 (37.1) |
| R-free (%) | 22.2 (37.1) |
| Number of non-hydrogen atoms | 19160 |
| macromolecules | 17840 |
| ligands | 4 |
| solvent | 1316 |
| Protein residues | 2269 |
| RMS (bonds, Å) | 0.003 |
| RMS (angles, °) | 0.6 |
| Ramachandran favored (%) | 96.7 |
| Ramachandran allowed (%) | 3.2 |
| Ramachandran outliers (%) | 0.2 |
| Rotamer outliers (%) | 0.16 |
| Clashscore | 3.16 |
| Average B-factor (Å^2^) | 40.8 |
| macromolecules | 40.7 |
| ligands | 36 |
| solvent | 41.7 |
